# Effects of dietary fish to rapeseed oil ratio on steatosis symptoms in Atlantic salmon (*Salmo salar* L) of different sizes

**DOI:** 10.1038/s41598-024-68434-3

**Published:** 2024-08-03

**Authors:** D. Siciliani, A. Hubin, B. Ruyter, E. M. Chikwati, V. G. Thunes, E. C. Valen, A. K. G. Hansen, H. Hanssen, T. M. Kortner, Å. Krogdahl

**Affiliations:** 1 Faculty of Veterinary Medicine, Norwegian University of Life Sciences, Ås, Norway; 2 Faculty of Chemistry, Biotechnology, and Food Sciences, Norwegian University of Life Sciences, Ås, Norway; 3 NOFIMA AS, Ås, Norway; 4 Biomar AS, Havnegata 9, 7010 Trondheim, Norway; 5 LetSea AS, Sandnessjøen, Norway

**Keywords:** Fat metabolism, Statistics

## Abstract

Choline is recognized as an essential nutrient for Atlantic salmon at all developmental stages. However, its dietary requirement is not well defined. Choline plays a critical role in lipid transport, and the clearest deficiency sign is intestinal steatosis. The present work, aiming to find whether lipid source and fish size may affect steatosis symptoms, was one of a series of studies conducted to identify which production-related conditions may influence choline requirement. Six choline-deficient diets were formulated varying in ratios of rapeseed oil to fish oil and fed to Atlantic salmon of 1.5 and 4.5 kg. After eight weeks, somatic characteristics were observed, and the severity of intestinal steatosis was assessed by histological, biochemical, and molecular analyses. Fatty acid composition in pyloric intestine, mesenteric tissue, and liver samples was also quantified. The increasing rapeseed oil level increased lipid digestibility markedly, enhancing lipid supply to the fish. Moreover, small fish consumed more feed, and consequently had a higher lipid intake. In conclusion, the results showed that choline requirement depends on dietary lipid load, which depends on the fatty acid profile as well as the fish size.

## Introduction

In recent decades, reduction in fish meal and fish oil in Atlantic salmon feeds has changed the dietary level of several essential nutrients. The consequences for the fish of such alterations are difficult to predict, since present knowledge concerning nutrient requirements in Atlantic salmon has severe gaps. It is highly likely that today’s feeds are suboptimal i.e., deficient or excessive, for some essential nutrients. Field observations of severe fat accumulation in the pyloric caeca of farmed Atlantic salmon, a condition known as intestinal steatosis, have recently been linked to deficient dietary supply of choline^1–3^. A mild degree of this condition seems to be a ubiquitous and underreported condition in cultivated Atlantic salmon, whereas cases of severe steatosis, categorized as lipid malabsorption syndrome (LMS), have also been reported^4^.

The investigations conducted to explore potential explanations for LMS led to confirmation of choline (C_5_H_14_NO_+_) as an essential nutrient for Atlantic salmon^1^. Choline has been acknowledged as essential for some fish species and other animals, particularly at early developmental stages^3,5–7^. Among its several functions, choline serves as a constituent of phosphatidylcholine, acetyl choline, and other cell components. In addition, as confirmed by a recent study conducted by Dhanasiri et al. 2024^8^, it plays a role in DNA methylation, by being an indirect methyl group donor for production of S-adenosylmethionine (SAM) during methylation processes.

Phosphatidylcholine, a class of phospholipids composed of a choline head group and glycerophosphoric acid, is a major membrane constituent of all living cells^5^. Phosphatidylcholine also plays a critical role in lipid transport and absorption by being an essential component of lipoproteins. Lipoproteins facilitate lipid transport from the intestine to the systemic circulation, as well as in the formation and secretion of very-low-density lipoprotein (VLDL) from hepatocytes^9^.

Symptoms of choline deficiency vary between fish species. The most apparent symptoms are related to impaired fat transport, comprising symptoms such as intestinal and hepatic steatosis, low growth rate, poor feed efficiency, and early death. Such symptoms have been observed in several cultivated species, including carp (*Cyprinus carpio*)^10^, lake trout (*Salvelinus namaycush*)^11^, rainbow trout (*Oncorhynchus mykiss*)^12^, yellow perch (*Perca flavescens)*^13^, channel catfish (*Ictalurus punctatus)*^14^ cobia (*Rachycentron canadum*)^14^, and, most recently, in Atlantic salmon (*Salmo salar L)*^3^*.*

Typical signs of intestinal steatosis in Atlantic salmon are pale and swollen appearance of the pyloric caeca and hyper-vacuolization of the tissue caused by the excessive lipid accumulation within the enterocytes. In extreme cases lipid absorption is severely reduced, and ingested lipid may accumulate all along the intestinal tract, to be eventually discarded as whitish faeces, a condition known as steatorrhea^1^.

According to present nutrient requirement guidelines, mostly based on observations of growth and hepatic steatosis, choline requirement of various fish species range between 400 and 1000 mg/kg feed. However, the work of Hansen et al.^3^ indicated that steatosis in the pyloric intestine is a more sensitive biomarker for estimating choline requirement. Hansen’s dose–response feeding trial^3^ indicated a choline requirement of 3400 mg/kg in Atlantic salmon kept in seawater and weighing 200-400g. The estimation was based on the observation of several biomarkers of lipid storage and transport, such as the organo-somatic indices of the pyloric and mid intestine, the histological evaluation of lipid vacuolation, as well as the expression levels of four genes with key roles in lipid metabolism, i.e., *apoA-IV, apoA-I, pcyt1α* and *plin2*.

The role of choline in lipid transport suggests that its dietary requirement may depend on several production-related conditions, such as life stage, size, growth rate of the fish, dietary lipid level and source, and environmental temperature. The present study was one of a series of screening studies underway to understand which biotic and abiotic conditions might affect steatosis symptoms and therefore choline requirement. Such knowledge would identify significant shifts in requirement level, to be taken into account when formulating optimal diets for fish farmed under the most demanding conditions. The aim of the present trial was therefore to understand whether fish size, and the dietary ratio of fish oil to rapeseed oil can affect the severity of the symptoms of induced steatosis. Findings will be used to attain indications over choline requirement in Atlantic salmon. A secondary aim was to strengthen understanding of fatty acid metabolism and transport in and between the intestine, mesenteric tissue, and liver.

## Materials and methods

With the aim of reducing the number of experimental animals, and for cost and time reasons, the current experiment was designed as a screening study. Therefore, we first observed the effects of choline deficiency on steatosis biomarkers in fish of two sizes, fed diets varying in fish oil to rapeseed oil ratios. Then, to achieve an indication of the quantitative aspects of the observed variation, we compared our data to the corresponding variation observed in Hansen et al.^3^’s regression study. In Hansen et al.^3^’s experiment, intestinal steatosis was induced by variation of dietary choline level, and the effects of such variation on the selected steatosis biomarkers were used to estimate choline requirement. In light of the differences between the present and Hansen et al.^3^’s study, the indicated changes in choline requirement do not represent an exact estimation, but they should merely be considered as indications.

### Feeds

Six experimental feeds were formulated varying in ratios of rapeseed to fish oil, from 0:31% to 24:8%. The nutritional content was, otherwise, similar. Feed ingredients and nutrient composition are shown in Tables 1 and 2. The average dietary choline amount was 1523 mg/kg, a level which, according to choline requirements set by Hansen et al.^3^, is severely deficient. This was a deliberate and necessary condition to evaluate the effects of specific factors on choline requirement. Yttrium oxide (Y_2_O_3_) was added as an inert marker to estimate apparent nutrient digestibility. The experimental feeds were produced by extrusion (feed pellet size 9mm) at BioMar Feed Technology Centre (Brande, Denmark) using a BC 45 twin-screw extruder (Clextral, France).

**Table 1:** Feed ingredients and nutritional composition of the experimental feeds.

	Rapeseed oil level, %					
	0	5	9	14	19	24
Ingredients, %						
Fish Meal NA LT	13.0	13.0	13.0	13.0	13.0	13.0
Krill 56	2.0	2.0	2.0	2.0	2.0	2.0
Soya SPC	23.8	23.8	23.8	23.8	23.8	23.8
Pea protein 65	7.5	7.5	7.5	7.5	7.5	7.5
Guar meal	8.0	8.0	8.0	8.0	8.0	8.0
Wheat milling quality	13.5	13.5	13.5	13.5	13.5	13.5
Fish oil	31.2	26.5	21.8	17.1	12.4	7.7
Rapeseed oil	0.0	4.7	9.4	14.1	18.8	23.5
Choline chloride 70%, dry	0.03	0.03	0.03	0.03	0.03	0.03
Vitamin and mineral mix*	2.1	2.1	2.1	2.1	2.1	2.1
Lucantin Pink CWD 10%, BASF	0.1	0.1	0.1	0.1	0.1	0.1
Yttrium oxide	0.1	0.1	0.1	0.1	0.1	0.1
Analyzed content						
Crude protein, %	38.6	38.3	38.6	38.3	39.7	39.6
Sum Fatty acids**, %	26.3	26.5	26.3	26.3	27.7	28.1
Choline, mg/kg	1490	1520	1680	1430	1500	1520

*The feeds were supplemented with standard vitamin and mineral premixes following NRC guidelines (2011) and BioMar standards to meet the requirements.

**See Table 2 for details.

**Table 2:** Fatty acid composition of the experimental feeds, % of diet.

	Rapeseed oil level					
Fatty acid	0	5	9	14	19	24
12:0	0.02	0.02	0.02	0.02	0.01	0.01
14:0	1.88	1.58	1.32	1.02	0.87	0.60
16:0	5.24	4.62	4.08	3.47	3.24	2.72
18:0	1.28	1.18	1.08	0.99	0.97	0.89
20:0	0.25	0.25	0.23	0.21	0.21	0.21
22:0	0.18	0.20	0.18	0.20	0.21	0.22
24:0	0.04	0.04	0.04	0.04	0.05	0.05
16:1	1.91	1.59	1.32	1.06	0.88	0.62
18:1	3.03	4.99	6.68	8.21	10.15	11.87
20:1	0.42	0.42	0.42	0.41	0.43	0.45
22:1n9	0.07	0.08	0.09	0.10	0.12	0.16
22:1n11	0.27	0.24	0.22	0.20	0.19	0.18
24:1	0.12	0.10	0.08	0.08	0.07	0.06
18:2n6	1.04	1.90	2.56	3.30	4.02	4.65
18:3n3	0.22	0.53	0.78	1.03	1.32	1.60
18:3n6	0.06	0.06	0.05	0.04	0.03	0.03
20:3n3	0.02	0.01	0.01	0.01	0.01	0.01
20:3n6	0.05	0.05	0.03	0.03	0.02	0.01
20:4n6	0.29	0.24	0.20	0.16	0.13	0.09
20:5n3	3.79	3.16	2.55	2.03	1.70	1.17
22:5n3	0.44	0.37	0.28	0.24	0.19	0.13
22:6n3	1.63	1.38	1.11	0.92	0.80	0.59
Other*	4.03	3.49	2.98	2.55	2.11	1.80
Tot sum	26.3	26.5	26.3	26.3	27.7	28.1

*Including unidentified fatty acids, and cis and odd chain fatty acids.

### Fish and rearing conditions

The feeding trial was conducted at the LetSea research facility in Dønna, Norway. Such facility is approved by The Norwegian Animal Research Authority (NARA), and it operates according to the Norwegian Regulations of 17th of June 2008 No. 822: Regulations relating to Operation of Aquaculture Establishments (Aquaculture Operation Regulations). Trial fish were treated in accordance with the Aquaculture Operation Regulations during the experiment. All the reported methods are in accordance with ARRIVE (Animal Research: Reporting of In Vivo Experiments) guidelines. As no harmful procedures were forced upon the fish before euthanasia, a specific permission was not needed for this experiment. Twelve steel-cages with a 125 m^3^ (5 × 5 × 5 m) volume, each fitted with a standard net and equipped with a lift-up system for feed waste collection, were used during the trial.

Atlantic salmon (*Salmo salar* L.) of two sizes were used, with average initial weights of 1.7 and 4.5 kg (CV = 10%), respectively. As it is not possible to produce fish with the same background but different size, it was decided to compare fish produced from autumn (S0/Small) and spring smolt (S1/Large). The small fish, delivered by AquaGen AS, were smoltified and put to sea in autumn 2020, while the large fish, delivered from Salmobreed AS, were smoltified and put to sea in spring 2020. Accordingly, the observed differences between the small and the large fish reflects the combined effects of fish size, fish breed and smoltification strategy.

The fish were pit tagged and randomly distributed to one of the six pens allocated for each size, 100 fish per pen. The feeds were randomly assigned to the pens. The fish were fed weighed amounts of feed twice a day, in excess. Uneaten feed was collected from a net under the pens for feed waste estimation. The feeding period lasted 55 days, from the 2nd of Sep to the 25th of Oct 2021, during which the fish were exposed to natural daylight and temperature conditions. Water salinity varied between 29 and 33 and the temperature ranged from 12 to 9 °C and averaged at 10.5 °C. Dissolved oxygen in the cages was measured daily, averaging at 80% throughout the experiment. Pictures showing the fish during the experimental set up are presented in Supplementary Figure S1a, b and c.

### Sampling

At the end of the feeding period, twelve fish were randomly taken from each pen using a land net before being anesthetized by exposure to tricaine methane-sulfonate (MS-222) at a concentration of 100mg/L for a minimum of 5 min. After recording final body weight (FBW, g) and fork length (FL, cm) of the fish, the blood was sampled from the caudal vein and stored on ice. Blood was then centrifugated and the obtained plasma was collected in 2mL aliquots, frozen in liquid nitrogen and kept at − 80 °C. Following blood sampling, the fish were killed by a sharp blow to the head, in accordance with the Norwegian Animal Welfare act and opened ventrally. The whole gastrointestinal package was removed from the abdominal cavity and the gutted carcass was weighed. Fish without feed content along the gastrointestinal tract were discarded. The intestine was sectioned into three parts^15^: pyloric intestine (PI), which extends from the pyloric sphincter to the most distal pyloric caecum; mid intestine (MI), which is the area between the latter pyloric caecum and the beginning of the visible complex mucosal folds of the distal intestine; distal intestine (DI), comprised between the MI and the anus. In the present study no observations were made on the MI section. A sample of the mesenteric fat from the PI area was collected. The three gut sections were opened longitudinally, and the digesta collected, snap frozen in liquid N_2_ and stored at − 80 °C until further enzyme activity analyses. The tissues sections were weighed, and samples collected. Tissue samples for histological analyses were first fixed in 10% neutral buffered formalin (4% formaldehyde) for 24 h, and subsequently transferred to 70% EtOH for storage until processing. Samples for RNA extraction were rinsed in sterile saline water, submerged in RNAlater®, incubated at 4 °C for 24h, and subsequently stored at − 20 °C until analysis. The remainder of each tissue section was frozen in liquid N_2_ and stored at − 80 °C for further enzyme activity analysis. The remaining fish in each pen were weighed individually. Estimates of growth performance, feed intake and mortality represent all fish in each pen.

### Welfare assessment

At the end of the feeding trial ten of the remaining fish were sampled randomly from each pen for welfare scoring (render score). At first, the number of Salmon lice (*Lepeophtheirus salmonis*) on each fish was counted. The parasites were classified based on their observed life stage as non-motile, Chalimus, motile and adult female. Lice released from the fish during anesthetization were counted and added to the total count for every fish. Considering the size difference, the number of lice was calculated per cm^2^ of fish surface. The surface area of the fish was calculated based on their weight following the formula of O’Shea et al.^16^. Thereafter, fish were evaluated for external indicators such as scale loss, skin lesions, eye damage and cataract, according to the scoring system developed by Kolarevic et al.^17^. Each fish was evaluated for every welfare indicator by assigning a score between 0 and 2, where 0 represents a good condition, while 2 stands for a bad condition. The dorsal and caudal fin evaluation was conducted using the scoring system developed by Hoyle et al.^18^ where the level of visible damages ranges from 0, no damages, to 5, complete erosion of the fin. Mortality was also used as a welfare indicator and the number of dead fish was collected along the feeding trial.

### Histology

The tissue samples collected from the pyloric caeca and the DI of the twelve fish sampled from each tank, were processed at the Norwegian University of Life Sciences (NMBU) using standard histological techniques: dehydration in graded ethanol, clarification in xylene, embedding in paraffin, and sectioning of 5 µm thick sections. The sections were further dewaxed, re-hydrated, stained with haematoxylin and eosin, and scanned using a Philips Ultra-Fast Scanner controlled using the Image Management System version 3.3 within the Philips IntelliSite Pathology Solution version 3.2 (Philips, Norway). Scans were then randomized and evaluated through a PC screen. The selected histological variables were submucosal infiltration of the DI and enterocytes steatosis in the PI. Concerning submucosal infiltration, the appearance of the DI was assessed based on those features which are characteristic of soybean meal-induced enteritis, respectively: changes in mucosal fold height and width, cellularity of the submucosa and lamina propria, and supranuclear vacuolization in the enterocyte^19^. A scoring system with a scale of 0–4 was used where 0 represented a normal condition, and 1 to 4 represented mild, moderate, marked and severe changes, respectively. The severity of steatosis was scored according to the proportion of tissue affected by the presence of lipid-like vacuoles, swollen and irregular cells and condensed nuclei, as Normal (≤ 10%), Mild (10–25%), Moderate (25–50%), Marked (≥ 50%) and Severe (≥ 75%) as shown by Siciliani et al.^20^.

### RNA extraction, cDNA synthesis and gene expression analyses

Gene expression analysis was performed on 144 tissue samples collected from the pyloric caeca. Treatments were randomized before the procedures started. RNA was extracted from pyloric caeca samples weighing 20–30 mg using an Precillus homogenizer, TRIzol® reagent (Invitrogen, ThermoFisher Scientific), and chloroform according to the manufacturer’s protocol. RNA was extracted with PureLink RNA mini kit (Invitrogen, ThermoFisher Scientific), and treated on column with PureLink DNase (Invitrogen, ThermoFisher Scientific). From each sample 2 µl were analysed in a NanoDrop ND-1000 Spectrophotometer (NanoDrop Technologies) to assess the RNA purity and concentration. The integrity of the RNA was verified on selected samples with a 2200 TapeStation (Agilent Technologies). Total RNA was then stored at − 80 °C for further analyses. Before cDNA synthesis RNA samples were pooled three by three according to their origin tank. Afterwards, 1 µg of each RNA pool and Superscript IV VILO (Invitrogen, ThermoFisher Scientific) in 20 µL reactions were used to conduct the cDNA synthesis. The same process was performed to achieve negative controls, omitting RNA and enzymes. cDNA was then diluted at 1:10 and stored at − 20 °C. The primers used in the qPCR reactions were obtained from literature and previous works conducted in the research group^1,3^,. Additional information concerning gene names, primer source, efficiency, and size, is shown in Supplementary Table S1. The efficiency (E) of the PCR reaction was assessed for each gene assay using serial dilutions of a pool of randomly selected cDNA samples. Additionally, when required, primer optimization was carried out by testing selected primer pairs at a range of temperatures in a single reaction. A LightCycler LC96 (Roche Diagnostic) was used to perform DNA amplification and gene expression analyses. Each reaction mix contained 2 µl PCR-graded water, 5 µl of LightCycler 480 SYBR Green I Master mix (Roche Diagnostics), and 0.5 µl of both forward and reverse primer. Every sample was analyzed in duplicate alongside a no-template control. The three-step qPCR program featured a first enzyme activation at 95 °C for 5 min, a following 40–45 cycles of 95 °C (10 s), 55, 58, 60, or 63 °C (10 s, depending on the single gene), and 72 °C (15 s). Quantification cycle (Cq) values were calculated using the second derivative method. The specificity of the qPCR reactions was confirmed by evaluating the melting curve of qPCR products and the band pattern on the agarose gel after electrophoresis. RNA polymerase II (*rnapoll*), hypoxanthine phosphoribosyl transferase 1 (*hprt1)* and glyceraldehyde- 3-phosphate dehydrogenase (*gapdh*) were evaluated for use as reference genes according to their stability across and within the treatments^21^. Target gene expression was normalized to the geometric mean of *rnapoll*, *hprt1* and *gapdh*. Mean normalized expression of the target genes was calculated from raw Cq values by relative quantification. The target genes selected to be assessed in this study correspond to the same set of genes involved in lipid transport and metabolism that Hansen et al.^3^ and Siciliani et al.^20^ identified as more receptive to steatosis symptoms and therefore to choline deficiency: *plin2, apoA-I, apoA-IV and pcyt1α.*

### Chemical analyses

Feed and faeces samples were analyzed for dry matter (DM), ash, crude protein (CP), crude fat (CF), fatty acids and starch at LabTek, Norwegian University of Life Sciences, Ås, Norway. Dry matter was established by drying samples to a constant weight at 105 °C. Ash content was assessed by combustion at 550 °C^22^. Total nitrogen, identified as crude protein was analysed by the semi-micro-Kjeldahl method (Kjeltec-Auto System, Tecator, Höganäs, Sweden). Fatty acid composition was analysed with the FAME method described by O`fallon, J.V 30^23^. Gross energy was recorded using the Parr 1271 Bomb calorimeter (Parr, Moline, IL, USA). Analyses of Yttrium oxide content in feed and faeces were conducted by pre-digestion with concentrated ultrapure HNO_3_ at 250 °C using a Milestone microwave UltraClave III (Milestone Srl, Sorisole, Italy). Samples were then diluted (to 10% HNO_3_ concentration), and Yttrium oxide was determined by inductively coupled plasma optical emission spectrometry (ICP-OES analysis) with a PerkinElmer Optima 5300 DV (PerkinElmer Inc., Shelton, CT, USA).

### Calculations

Fish growth was estimated as thermal growth coefficient (TGC) = 1000 ∗ [ BW1^1/3^ − BW0^1/3^)/ddg] in which BW0 and BW1 are the initial and final body weight and ddg is daydegrees (no of feeding days (D) x average temperature in ◦C) and specific growth rate (SGR) = (ln BW1/ln BW0)/D) × 100. The condition factor (CF) was estimated as: CF = 100 x (FBW)/FL) (FL: (fork length (cm)^3^). The organ somatic indices (OSI) were calculated as: (organ weight g/body weight g) × 100). Apparent digestibility (AD) for each nutrient was determined by using Yttrium oxide as an inactive marker and estimated as follows: ADn = 100 − (100 × (Mfeed/Mfaeces) × (Nfeed/Nfaeces)), where M represents the percentage of Yttrium oxide in feed and feces and N represents the percentage of a specific nutrient in feed and feces. To estimate the fatty acid (FA) content in the diets and organs, tridecanoic acid (13:0) was used as internal standard and added to the samples, 0.25 mg per 500 mg sample. The following formulas were applied for the estimations: FA (mg) = (peak area FA/peak area 13:0) × RF (response factor) × 13:0(mg); and: FA (mg/g feed or tissue) = FA (mg)/weighted sample (g). The RF is proportional with the number of active carbons in the fatty acid chain and varies among different fatty acids. Content of digestible fatty acids in the diet was calculated as for other nutrients based on fatty acid in the faeces and in the diet employing level of Yttrium oxide in the faeces and diet.

### Statistical analysis

#### Histological observations

The scores for both submucosal infiltration (DI enteritis) and enterocyte steatosis were categorical variables, and the impact of fish size and rapeseed oil level on the distribution of the histological scores among the diet groups were explored by ordinal logistic regression run in the R statistical package (version 4.2.1; 2022) using the polr (proportional odds logistic regression) package within the RStudio interphase (version 2023.06.1 + 524). Differences were examined based on odds ratios of large fish and rapeseed oil level having different histology scores compared to the small fish and the 0%-rapeseed oil diet.

#### Welfare scores

Statistical analyses of scale loss, skin lesions, eye damage and cataract as well as the sea lice number were performed using the unpaired t-test and fish size was considered as main variable. All data are means ± SEM. The level of significance was set to *P* < 0.05.

#### Other results

For the main body of results a Bayesian approach was selected for the statistical analysis. This approach provides uncertainty awareness and robust reasoning for resource demanding studies aiming to describe dose–response relationships even when the number of observations is relatively small and without replications^24^. Although other approaches are available to define linear regressions functions from studies without replications^25^, the Bayesian represents the most reliable approach to the influence of the outliers, enabling proper handle uncertainty on the key parameters and uncertainty in the models^26^.

For simplicity, assume $n$ observations for every studied dependent variable, denoted as $y_{i} , i \in \left\{ {1, \ldots ,n} \right\}$ and the vector of corresponding independent variables ${\varvec{x}}_{i} .$ Further, for every studied dependent variable, 5 different models are suggested corresponding to 5 hypotheses on the dependence between the dependent and independent variables. More specifically $Y_{i} \sim N\left( {\mu_{k} \left( {{\varvec{x}}_{i} } \right),{\upsigma }^{2} } \right), k \in \left\{ {1, \ldots ,5} \right\}$ corresponding to models $m_{1} , \ldots ,m_{5}$ with:

1. $m_{1} :\mu_{1} \left( {{\varvec{x}}_{i} } \right) = \beta_{0}$—no effects of ${\varvec{x}}_{i}$ on $y_{i}$ (intercept only model).
2. $m_{2} :\mu_{2} \left( {{\varvec{x}}_{i} } \right) = \beta_{0} + \beta_{oil} RapeOil_{i}$—intercept and a linear dependence on $RapeOil_{i}$ variable.
3. $m_{3} :\mu_{3} \left( {{\varvec{x}}_{i} } \right) = \beta_{0} + \beta_{size} Size_{i}$—intercept and a linear dependence on $Size_{i}$ variable.
4. $m_{4} :\mu_{4} \left( {{\varvec{x}}_{i} } \right) = \beta_{0} + \beta_{size} Size_{i} + \beta_{oil} RapeOil_{i}$—intercept and a linear dependence on $RapeOil_{i}$ and $Size_{i}$ variables.
5. $m_{5} :\mu_{5} \left( {{\varvec{x}}_{i} } \right) = \beta_{0} + \beta_{size} Size_{i} + \beta_{oil} RapeOil_{i} + \beta_{size,oil} Size_{i} \times RapeOil_{i}$—intercept and a linear dependence on $RapeOil_{i}$ and $Size_{i}$ variables as well as the interaction effect between them.

Since models $m_{1} , \ldots ,m_{5}$ were addressed corresponding to 5 hypotheses on the relations between the dependent and independent variables to choose from, model uncertainty should also be carefully addressed. For the model priors $p\left( {m_{1} } \right), \ldots ,p\left( {m_{5} } \right)$, a uniform prior was used in the model space corresponding to all model priors being $p\left( {m_{1} } \right) = \ldots = p\left( {m_{5} } \right) = \frac{1}{5}$. This gives us a fully non-informative prior preference across the considered models. Further, default priors from *R-INLA*^27^ library are assumed for the parameters. These priors for the fixed effects are standard independent normal with the mean of 0 and the variance of 1000, i.e., $N\left( {0,1000} \right)$ and are quite flat and are very weakly informative. For the intercept, $N\left( {0,\infty } \right)$ priors were used, which are completely flat and uninformative. Such a choice of priors is meant to reduce subjectivity of the models. A Bayesian framework does not require fit tests explicitly as we have hypotheses corresponding to m1 with no effects at all and if the evidence coming from the data is not strong enough, we shall prefer this simpler m1 to more complicated models.

For inference, the integrated nested Laplace approximations^28^ implemented in *R-INLA* library were used to obtain the quantiles of all the marginal posterior distributions of interest. Also, for the Gaussian regression with Gaussian priors, exact marginal likelihoods $p\left( {{\varvec{yx}},m} \right)$,$m \in \left\{ {m_{1, \ldots ,} m_{5} } \right\}$ can be computed by *R-INLA* and allow to compute the marginal posterior model probabilities $p\left( {m{\varvec{x}},{\varvec{y}}} \right) = \frac{{p\left( {{\varvec{yx}},m} \right)p\left( m \right)}}{{\mathop \sum \nolimits_{{m^{\prime} \in \left\{ {m_{1, \ldots ,} m_{5} } \right\}}} p\left( {{\varvec{yx}},m^{\prime}} \right)p\left( {m^{\prime}} \right)}} ,m \in \left\{ {m_{1, \ldots ,} m_{5} } \right\}$ through renormalization and thus accurately account for model uncertainty in the post-selection inference. Since uniform model priors are used selecting the model with respect to posterior marginal probabilities coincides with the selection according to Bayes factors between models $m$^29^ and $m^{\prime}: BF\left( {m,m^{\prime}} \right) = \frac{{p\left( {{\varvec{yx}},m} \right)}}{{p\left( {{\varvec{yx}},m^{\prime}} \right)}}$. In this paper for each dependent variable Bayes factor between the most probable and second most probable models are reported as BF12.

Supplementary Table S2 explains terms and abbreviations used in the output from Bayesian statistics which are relevant for the present work.

The term Bayesian factor, BF12, is key to the interpretation of the results. In the notion of Kass and Raftery^29^, the values of Bayes factors indicate evidence for relationship as follows: Values between **1** and **3.2**: *Negligible*; between **3.2** and **10**: *Substantial*; Between **10** and **100**: *Strong*; > **100**: *Decisive*.

Most of the standardized residuals of the finally selected models showed satisfactory characteristics regarding the requirement for homogeneity in normality and variance according to the p-values of Kolmogorov–Smirnov test (KS)^30^ with the null hypotheses that standardized residuals come from a homoscedastic standard normal distribution, i.e. for KS > 0.05 we cannot reject the null, while for results showing KS < 0.05, the null is rejected, and interpretation of individual effects should be done with caution, although we still have valid conclusions from the marginal posterior model probabilities and Bayes factors on the explored set of models for each response.

Comparisons across models for different dependent variables described in the Results chapter were made based on visual examination of the graphs, considering results for which the 95% credible intervals are not overlapping as significantly different. These conclusions are only made for the models where the assumptions on the residuals are satisfied.

## Results

### Growth performance

Growth performances were overall good. Small fish grew on average 1.4 kg, reaching a final body weight of 3.1 kg, the large grew 1.9 kg and reached 6.4 kg, giving an averaged SGR of 1.04 and TGC of 4.3 for the small fish, and 0.63 and 3.4 for the large, respectively. See Supplementary Table S3 for details and explanation of parameters. The statistical analyses of the results for TGC showed decisive evidence for m3 (BF12 = 919; Model probability (Prob) = 0.999), meaning that fish size but not dietary lipid source affected the result decisively. Regarding FCR, which averaged 1.1 and 1.2, and CF, which averaged 1.50 and 1.52, respectively, the analyses showed decisive evidence for m1 (BF12: 5157 and 2408; Prob: 1.000 and 0.999, respectively), meaning that neither fish size nor dietary lipid source affected these variables importantly. Feed consumption over the observation period, expressed as percent of initial body weight, was 90% for the small and 42% for the large, meaning a much higher feed and lipid load for the small than the large fish.

### Nutrient digestibility

Results regarding nutrient digestibility, best model, probabilities, and BF12, are shown in Table 3 and 4, whereas further statistical characteristics are presented in Supplementary Table S4. The statistics indicated high model probability for all observed nutrients, i.e., *p* > 0.844, and correspondingly high BF12, i.e., > 9.6 (8 nutrients showed substantial evidence levels, 14 nutrients showed strong evidence levels, and 1 decisive level). The exceptions were 16:1 and 18:2 which showed lower model probabilities. Regarding digestibility of crude protein, the evaluation showed substantial evidence for model m3 (BF12: 6; Prob: 0.863), i.e., effect of fish size but not of increasing rapeseed oil level and decreasing fish oil in the feed. For digestibility of fatty acids, the evaluation only indicated effects of increasing rapeseed oil level, and not of fish size, i.e. m2, for most, but not all, as follows: For the saturated fatty acids, as well as 20:1, 22:1, 18:2 and 18:3, evidence for m2 was strong, whereas the digestibility of the polyunsaturated fatty acids were not affected by rapeseed oil level (best model: m1).

**Table 3:** Digestibility (%) of crude protein (CP), Sum of fatty acids (FA), saturated and mono-unsaturated given as averages of small and large fish.

	CP	Sum FA	14:0	16:0	18:0	20:0	16:1n7	18:1n9	20:1	22:1n9
Rapeseed oil level										
0	86.8	81.0	75.0	60.5	41.5	52.5	96.5	93.0	89.5	80.0
5	86.2	81.0	73.0	58.5	37.5	50.5	95.0	93.5	88.0	81.5
9	88.1	87.5	81.0	68.5	47.0	56.0	98.0	97.0	94.0	89.0
14	86.1	89.0	82.0	70.5	48.0	56.5	98.0	97.0	93.5	91.0
19	87.8	92.0	87.5	79.0	57.0	64.0	98.5	98.0	95.5	94.0
24	86.6	94.0	91.0	85.0	63.5	70.0	98.5	98.0	95.5	95.5
Statistics										
Best model	m3	m2	m2	m2	m2	m2	m1	m1	m2	m2
Probability	0.863	0.961	0.944	0.922	0.886	0.844	0.901	0.473	0.530	0.929
BF12	6	25	18	12	10	10	12	1	1	14
Model evidence	Subst	Strong	Strong	Strong	Strong	Strong	Strong	Negl	Negl	Strong

**Table 4:** Digestibility of n-6 and n-3 fatty acids.

	18:2n6	20:4n6	18:3n3	20.5n3	22:5n3	22:6n3
Rapeseed oil level						
0	89.0	97.0	90.0	98.0	97.0	96.0
5	92.5	96.5	94.5	97.5	95.0	95.0
9	96.0	98.5	97.0	99.0	97.5	97.0
14	96.0	98.0	97.5	99.0	97.5	97.0
19	97.0	98.0	98.0	99.0	97.5	97.0
24	97.0	97.5	98.5	99.0	96.5	96.5
Statistics						
Best model	m2	m1	m2	m1	m1	m1
Probability	0.893	0.902	0.893	0.944	0.804	0.928
BF12	12	9	14	19	4	13
Model evidence	Strong	Subst	Strong	Strong	Subst	Strong

Lipid digestibility in quantitative terms: the digestibility of sum of fatty acids was greatly affected by lipid source. Indeed, digestibility increased of 13%-units as dietary rapeseed oil level increased from 0 and the 24% in the feed. The greatest elevating effects were seen for saturated fatty acids, with a difference between the feed with the lowest and highest rapeseed oil level of more than 20%. The longer and more unsaturated fatty acids showed higher digestibility, but still with clear, elevating effect of rapeseed inclusion level for 20:1 and 22:1, 18:2, and 18:3. The acids 16:1 and 18:1 showed the same trend, whereas the very long an unsaturated fatty acid appeared unaffected by rapeseed oil level in the feed. See Supplementary Table S4 for further statistical details.

### Plasma biochemistry

The observed plasma biomarkers showed decisive evidence for model m3 for alanine amino transferase (ALT), i.e., showing effects of fish size, but not of increasing rapeseed oil and decreasing fish oil level. The ALT averaged 25 and 17 U/l for the small and the large fish, respectively. The other observed plasma biomarkers showed no clear effects neither of fish size nor of lipid source, i.e., m1 was the best model. The observed averages were for free fatty acids (FFA) 0.32 mmol/l, glucose (Glu) 6.8 mmol/l, cholesterol (Chol) 7.5 mmol/l, and for triglycerides (TG) 2.7 mmol/l. See Table S5 for statistical details.

### Organ indices and lipid content

Results regarding organosomatic indices (OSI) of the PI, DI, Mes, and LI, as well as lipid concentration in PI, Mes, and LI are shown in Table 5 (See Supplementary Tables S3 and S5 for further details and statistics). The identified best models showed high probability with correspondingly high BF12, i.e., above 44, characterized as decisive evidence, for all these results. The exception was lipid level in Mes and liver which showed lower best model probability.

**Table 5:** Results for sampled fish regarding effects of fish size (0 = small, 1 = large) and feed rapeseed oil level on sum of identified digestible fatty acid (∑Dig. FA), somatic index (% of body weight) of pyloric intestine (OSIPI) cleaned of mesenteric tissue (Mes), distal intestine (OSIDI), Mes (OSIMes), and liver (OSILI), as well as lipid content (Sum of fatty acids, g/kg tissue) of PI, Mes, and LI (PI Lipid, Mes Lipid, LI Lipid).

		∑dig. FA	OSIPI	PI Lipid	OSIDI	OSIMes	Mes Lipid	OSILI	LI Lipid
		g/kg feed	%	g/kg	%	%	g/kg	%	g/kg
Fish size	Rapeseed oil level								
0	0	189	2.6	66	0.5	6.2	610	1.21	34
0	5	197	2.9	90	0.4	6.6	589	1.27	30
0	9	207	2.6	77	0.5	6.3	830	1.26	35
0	14	214	2.8	107	0.5	6.5	688	1.33	35
0	19	238	2.7	121	0.5	6.5	726	1.39	46
0	24	251	2.9	129	0.5	6.6	648	1.29	55
1	0	181	2.0	69	0.6	6.4	633	1.23	27
1	5	187	1.9	62	0.5	6.3	694	1.27	29
1	9	211	2.3	84	0.4	7.0	736	1.23	30
1	14	217	2.2	66	0.5	6.8	697	1.29	32
1	19	242	2.4	87	0.5	7.3	796	1.26	32
1	24	250	2.3	97	0.5	6.7	728	1.32	36
Statistics									
Best model		m2	m3	m4	m1	m1	m4	m1	m4
Probability		0.919	0.978	0.77	0.921	0.999	0.459	0.999	0.436
BF12		11	45	7	12	898	2	2161	2
Model evidence		Strong	Strong	Strong	Strong	Decisive	Negl	Decisive	Negl

For lipid concentration in the PI tissue, model m4 was the best, showing a clear, increasing effect of increasing rapeseed oil level, higher values for the small fish, as well as an interaction between the two fish sizes, i.e., the difference between the small and the large fish increased with increasing rapeseed oil level in the diet. For the OSIPI m3 was the best model, showing higher values for the small fish than the large, but no clear effect of dietary lipid source. Regarding the distal intestine (DISI), OSIMes and liver (LISI), no clear effects of lipid source or fish size were observed (best model: m1). Although the model evidence was low for Mes Lipid and LI Lipid, it is worth mentioning that m4 was the best model for both, i.e., indicating a trend of effect of increasing rapeseed oil level, fish size, as well as interaction between the two. Liver glycogen which averaged 1.9% of the liver tissue, seemed unaffected by rapeseed oil level as well as fish size (data not shown).

### Histology

**Figure 1:**
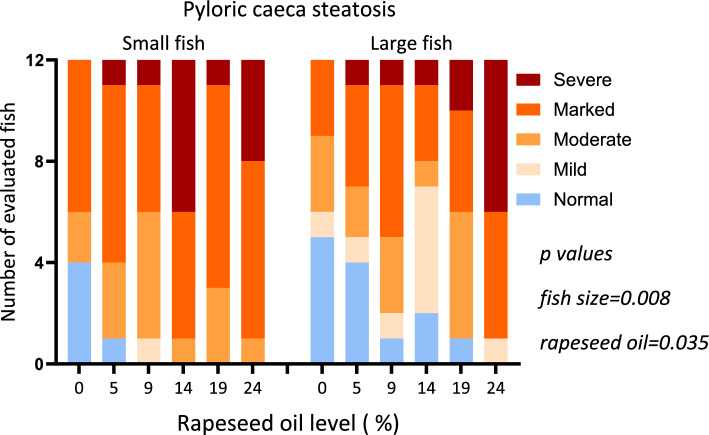
Distribution of the histology scores for enterocyte steatosis of the pyloric caeca tissue among the treatment groups. X-axis presents rapeseed oil level for the two fish sizes: small (S1 = autumn smolts) and large (S0 = spring smolts) fish. Table insert presents results for an ordinal logistic regression of impact of fish size and rapeseed oil level on the distribution of the histological scores among the diet groups for pyloric caeca enterocyte steatosis.

**Figure 2:**
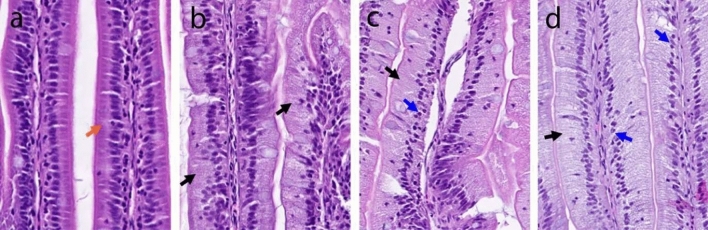
Representative images of the pyloric caeca mucosal folds showing a close-up appearance of the different scores for the enterocyte steatosis changes. a shows no enterocyte vacuolization (orange arrow) which is normal morphology, while images b, c, and d show enterocyte steatosis changes graded as moderate, marked, and severe, respectively. Black arrows in images b to d depict the lipid accumulation in the supranuclear space of the enterocytes while the blue arrow in images c and d show the progressive squashing of the enterocyte nuclei from the normal ellipsoid shape to a smaller and more spherical shape as intracellular lipid accumulation increases.

The histology scores showed significant effects of fish size (*p* = 0.008) on steatosis symptoms in the pyloric caecal enterocytes (Fig. 1). The severity of the symptoms increased significantly with increasing level of rapeseed oil and decreasing level of fish oil in the feeds (*p* < 0.035). Assigning a score of 0 for normal appearance, 1 for mild, 2 for moderate, 3 for marked and 4 for severe (Fig. 2) and fitting a first-degree linear regression line to the results, gives the following equations for the small fish: y = 0.0532x + 3.1111, for the large fish: y = 0.0699x + 2.3452. The equations give average score for small and large fish fed 0 rapeseed oil equal to 3.1 and 2.3, respectively, i.e., a difference of 0.8, and for fish fed 24% rapeseed oil of 4.3 and 4.0, i.e. a difference of 0.3. The functions indicate a difference in score between small fish fed 0 and 24% rapeseed oil of 1.2, and for the large fish a score of 1.7.

**Figure 3:**
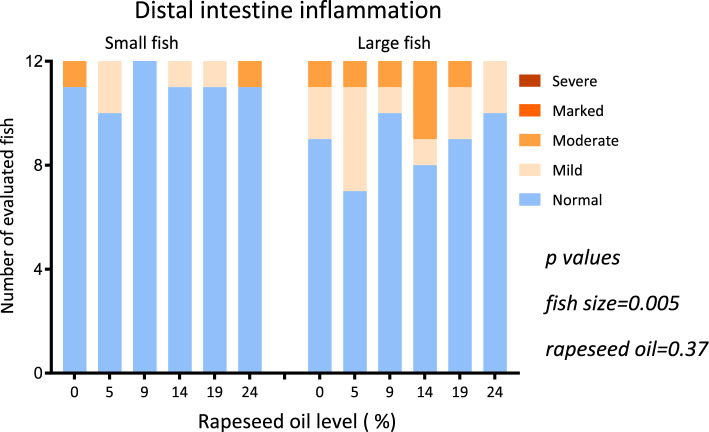
Distribution of histology scores for inflammatory cell infiltration in the submucosa and lamina propria of distal intestine among the treatment groups. X-axis presents rapeseed oil level for the two fish sizes: small (S1 = autumn smolts) and large (S0 = spring smolts) fish. Table insert shows results for an ordinal logistic regression of impact of fish size and rapeseed oil level on the distribution of the histological scores among the diet groups for distal intestine submucosal infiltration.

The histological evaluation of inflammatory markers in PC and DI showed that most of fish displayed normal and healthy morphology. Some individuals in all diet groups showed mild to moderate submucosal cellularity in the DI (Fig. 3). A significant effect of fish size was discerned (*p* = 0.005; Fig. 3) for occurrence of distal intestinal enteritis with more of the large fish observed with changes. No significant effects of dietary rapeseed oil level were observed on inflammatory changes (*p* = 0.37).

### Gene expression

**Figure 4:**
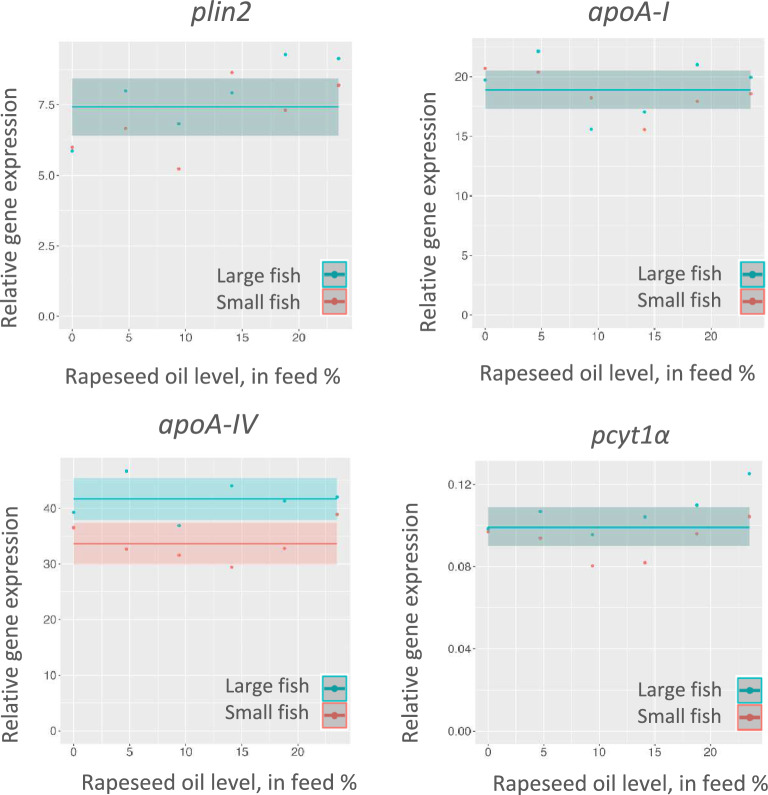
Relative expression of biomarker genes for choline requirement. The curves show the estimated regression lines with indication of 95% credible intervals for posterior mean.

The gene expression results (Supplementary Table S6) showed high probabilities and high values of BF12, respectively: strong for *apoA-I* and *apoA-IV,* and decisive and substantial for *pcyt1α* and *plin2*. In detail: for *apoA-I, pcyt1a and plin2* the best model was m1, meaning no clear effect of either fish size or increasing rapeseed oil level. The best model for *apoA-IV* was m3, showing effects of fish size, being more expressed in the large than in the small fish. However, no clear effects of rapeseed oil level were detected (Fig. 4).

### Fatty acid profile and content in feed, pyloric intestine, mesenteric adipose tissue, and liver

**Figure 5:**
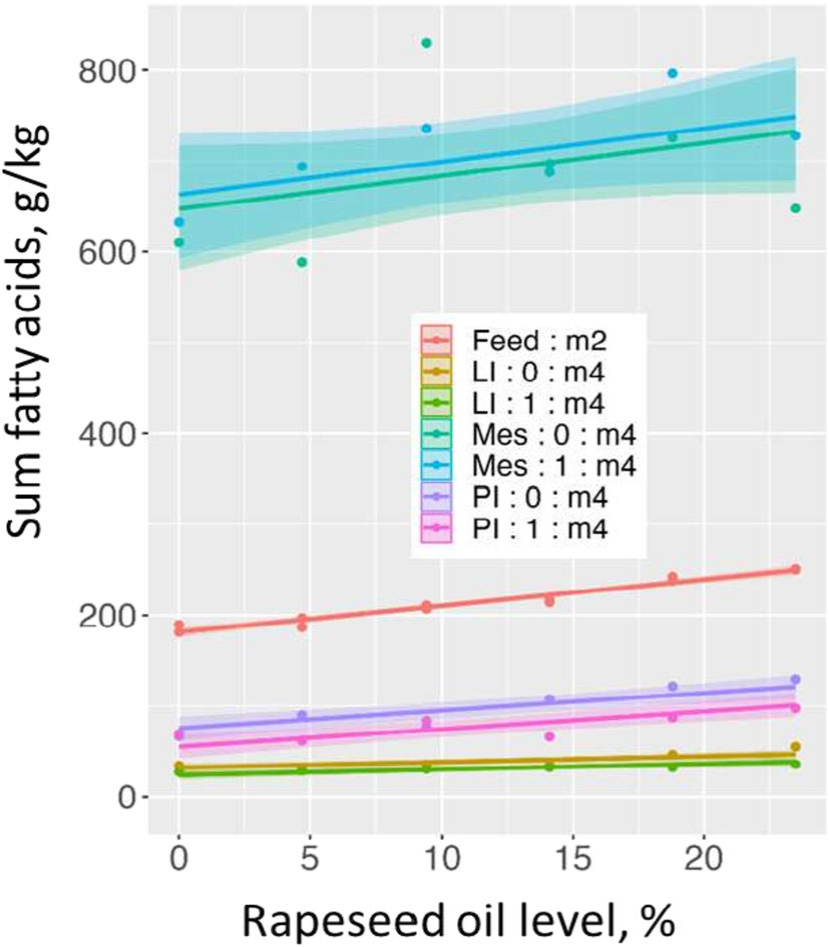
Effects of the increasing dietary rapeseed oil level on the sum of fatty acids in pyloric caeca (PI), liver (LI), mesenteric fatty tissue (Mes) and on the sum of absorbable fatty acids in the feed (Feed) (Unit: g/kg feed or tissue), for small (0) and large (1) fish. The legend in the figure presents the best model for the data. The curves show estimated regression on dietary rapeseed oil level with indication of 95% credible intervals for the posterior means, allowing comparison of the results: lines and parts of lines for which the 95% range do not overlap differ significantly.

**Figure 6:**
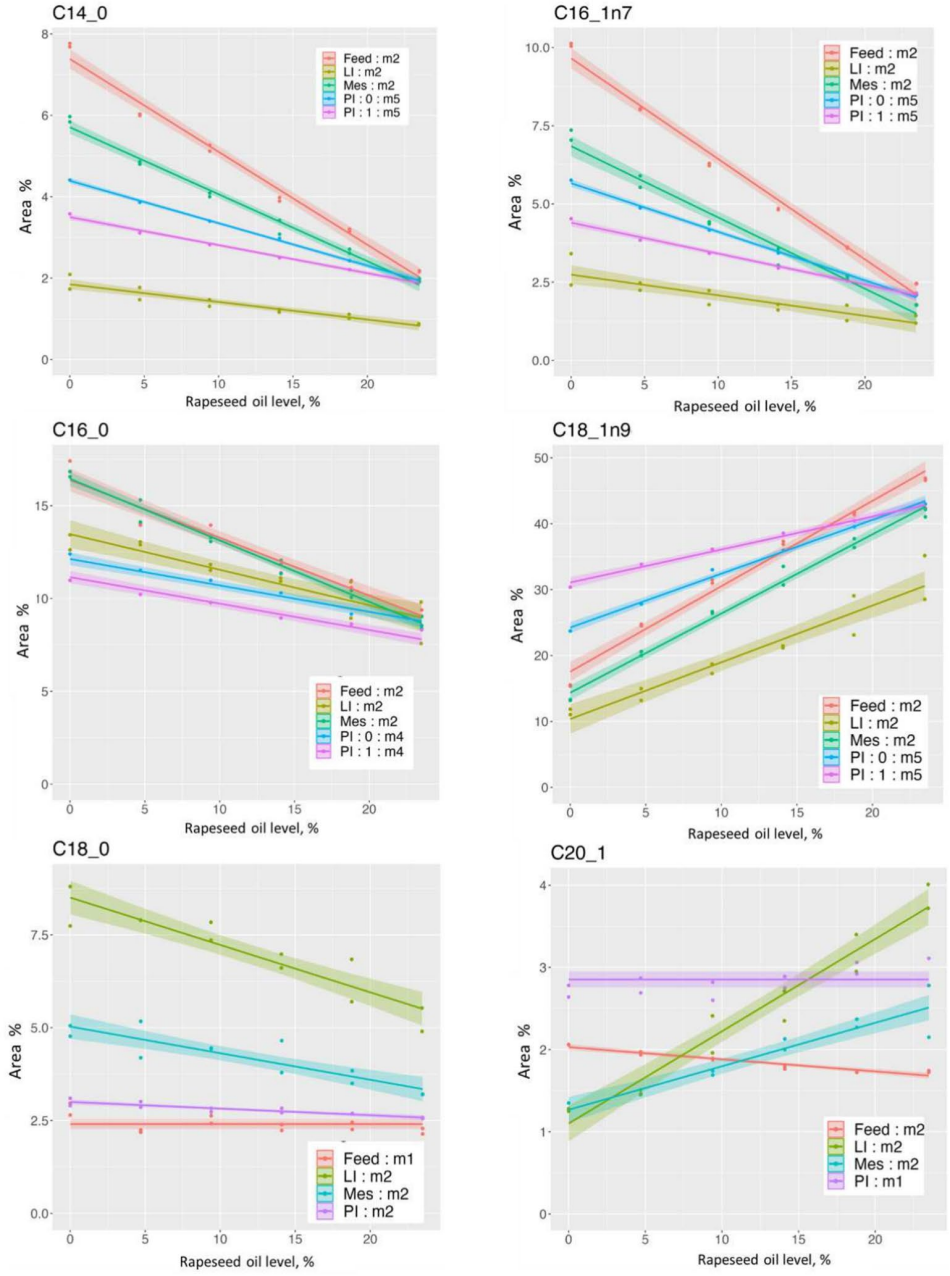

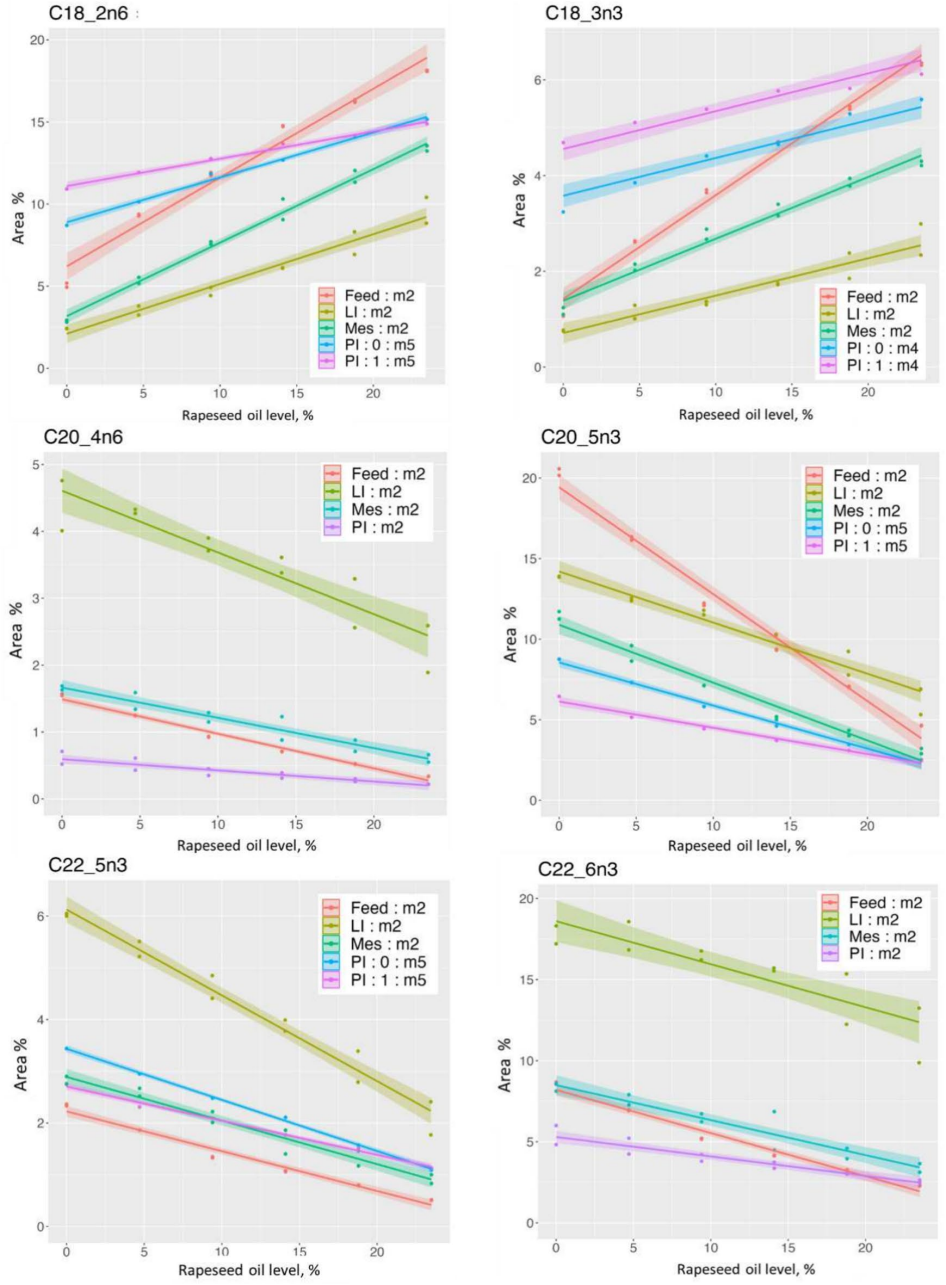
a. Effects of the increasing dietary rapeseed oil level on the relative level of saturated and mono-unsaturated fatty acids (% of sum of fatty acids, Area %) indicated on the left above the graphs, in absorbed fat, pyloric caeca (PI), mesenteric fatty tissue (Mes), and liver (Unit: % of sum fatty acids), representative for small (0) and large (1) fish. The legend in the figure indicates whether fish size clearly affected the results and the best model selected for the data. For fatty acids not clearly affected by fish size, average cures are presented. For fatty acids showing significant effects of fish size, separate curves are shown. The curves show the estimated regression lines with indication of 95% credible intervals for posterior means. The curves show estimated regression on dietary rapeseed oil level with indication of 95% credible intervals for the posterior means allowing comparison of the results: lines and parts of lines for which the 95% range do not overlap differ significantly. b Effects of the increasing dietary rapeseed oil level on the relative level (% of sum of fatty acids, Area %) of poly-unsaturated fatty acids indicated on the left above the graphs, in absorbed fat, pyloric caeca (PI), mesenteric fatty tissue (Mes), and liver (Unit: % of sum fatty acids), representative for small (0) and large (1) fish. The legend in the figure indicates whether fish size clearly affected the results and the best model selected for the data. For fatty acids not clearly affected by fish size, average cures are presented. For fatty acids showing significant effects of fish size, separate curves are shown. The curves show the estimated regression lines with indication of 95% credible intervals for posterior means. The curves show estimated regression on dietary rapeseed oil level with indication of 95% credible intervals for the posterior means allowing comparison of the results: lines and parts of lines for which the 95% range do not overlap differ significantly.

Table 5 and Fig. 5 show results regarding sum of fatty acids given as g digestible fatty acid/kg diet and g/kg tissue. Figure 6a and b show results regarding concentration of fatty acid in samples of the PI, Mes, and LI as well as absorbable fatty acid in the feed, given as % of sum of fatty acids for fatty acids present at levels > 1%. The fatty acid results expressed as g/kg diet or tissue are shown in Supplementary Figure S2a and b. All results of the fatty acid analyses, including statistics, are shown in Supplementary Table S7a and S7b. As described under the chapter Statistics in Materials and Methods, the comparisons presented below of the models for the different dependent variables were based on visual examination of the graphs, considering results for which the 95% credible intervals are not overlapping as significantly different, and conclusions are only made for the models where the assumptions on the residuals are satisfied.

Sum of digestible fatty acids in the diet and sum of fatty acids in PI, Mes, and LI, expressed in g/kg, increased with increasing rapeseed oil level in the diet. The Mes tissue was clearly a lipid storage organ, with more than 60% fat (589–830 g/kg). Lipid level in the PI was low (62–129 g/kg), but higher than of the liver (27–55 g/kg). Clear but minor effects of fish size were observed.

At low rapeseed oil level, the lipid fraction of the PI tissue, through which the digestible fatty acids enter the body, contained lower concentrations (% of sum of fatty acids) of 14:0, 16:0*, 16:1*, 20:4n6, 20:5n3*, and 22:6n3 compared to the absorbable fat in the feed. At higher levels of rapeseed oil the difference was less or absent. For the fatty acids marked with an asterix, evidence for effect of fish size was observed. For 18:0, 18:3n3*, and 22:5n3 the picture was opposite, with higher levels of such fatty acids in the PI than in the feed. Again, the difference was greatest at low rapeseed oil level except for 22:5n3 for which the difference did not differ with rapeseed oil inclusion. For 18:1*, 18:2n6*, 20:1, the level in PI was lower than in the feed at low rapeseed oil level, higher at high levels.

In Mes, the tissue supposedly first in line to receive fatty acids after crossing the intestine, 14:0, 16:0, 16:1, 20:4n6, 20:5n3, and 22:6n3 showed concentrations lower than in the diet but higher than in the PI. For 18:0 the distance to the diet observations was greater, i.e., the concentration was much higher than for PI, diminishing with increasing rapeseed oil level, whereas 18:3n3 was lower in the Mes than in the diet, increasing with increasing rapeseed oil level. For 18:1 and 18:2n6 the level in the Mes was lower than in PI, and lower than in the diet. 22:5n3 was lower than in the PI, but higher than in the diet, i.e., intermediate between the PI and the diet. 20:1 showed a mixed picture with lower levels than in PI for all rapeseed oil levels, and lower levels than in the diet at the low rapeseed oil inclusion level, higher at the high levels.

In LI, an organ playing a key role in metabolism of fatty acids after absorption and transport to the peripheral circulation, the evidence for model m2 was high for all fatty acids. The exceptions were 12:0 and 20:0, for which m1 showed high evidence, i.e., no effect of rapeseed oil level or fish size. For the fatty acids 14:0, 16:0, 16:1, 18:1, 18:2, and 18:3, the level in the liver was lower than in the absorbed lipid, for 18:0, 20:4, 22:5 and 22:6 it was higher, whereas for 20:1, 20:5 the level was quite similar.

### Welfare

The complete dataset of the welfare assessment is given in Supplementary Table S8. Among the 1200 fish used in the trial, 50 died during the feeding period, randomly distributed among the treatments. Sea lice infestation was a significant challenge for the fish during the experiment, as it is for most Norwegian salmon farms. The number of lice, recorded as motile lice as well as adult female lice, was significantly higher for the smaller fish than the large. Scale loss showed the same picture. There was no significant effect of rapeseed oil level on lice infestation or scale loss. The scores for the other observed welfare indicators, i.e., skin lesions, eye damage, cataract and fin erosion were not affected either of fish size or rapeseed oil level.

## Discussion

### Effects of fish oil to rapeseed oil ratio on choline requirement

While planning the current study, the idea was to investigate the effects of dietary lipid source on the severity of steatosis symptoms, by employing diets with constant lipid level and varying ratio of fish oil to rapeseed oil, i.e., the main marine and plant oil used in salmon diets. However, the variation in lipid source affected the lipid and fatty acid digestibility to a much greater degree than expected, and made the diets differ in total content of digestible lipid. The lipid digestibility of the diet without rapeseed oil was quite low, 81%. Similarly low digestibility coefficients have been observed in other studies conducted on Atlantic salmon. Karalazos et al.^31^, comparing diets varying in proportion of lipid sources, observed that the diet with the highest fish oil content was also the one showing the lowest lipid digestibility. On the other hand, increasing rapeseed oil level increased lipid digestibility. Another study, conducted by Ng et al.^32^, showed that varying the proportion of rapeseed oil and crude palm oil in salmon feeds caused a variation in lipid digestibility of 3% for total fat, and 11% for 16:0. In the present study, the increasing rapeseed oil level enhanced lipid digestibility and therefore dietary lipid supply to the fish. This observation should be seen in relation to the results obtained from our recent, related study, which showed that dietary lipid level is the main factor influencing choline requirement in Atlantic salmon^20^.

**Figure 7:**
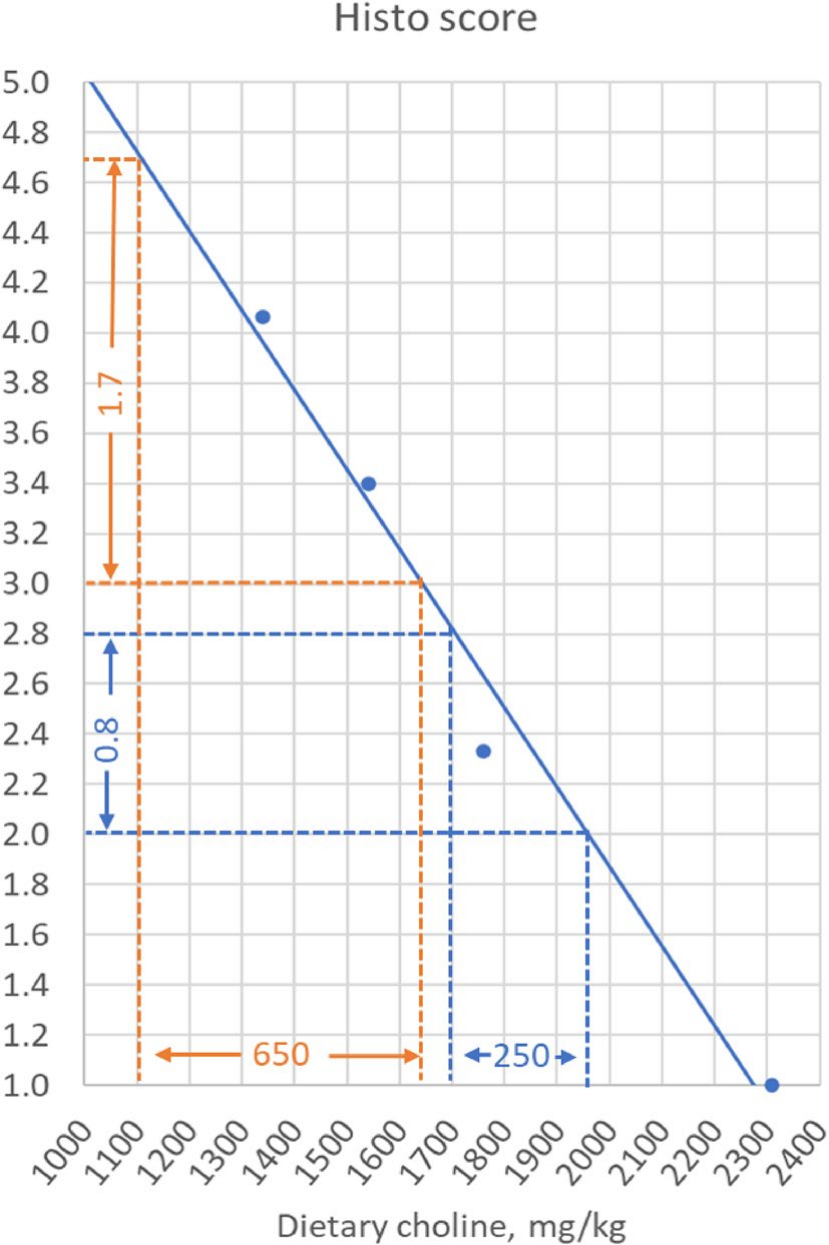
The curve in the figure illustrates the dose–response relationship between dietary choline level and intestinal steatosis, as previously published by Hansen et al.^3^. The dotted lines illustrate the strategy used for estimation of effect on choline requirement in the present study. The increase in rapeseed oil from 0 to 24% increased the score by 1.7 units (orange line) in the large fish. The figure indicates that this shift corresponds to a shift in choline requirement of about 650 mg/kg. The shift in the small fish was 1.2, corresponding to a shift of 450 mg/kg (not illustrated). The difference between the two fish sizes, when fed the lowest rapeseed oil level, was 0.8 units (blue line), i.e. lower in the small than the large fish, corresponding to a difference in choline requirement of about 250 mg/kg. At the highest rapeseed oil level, the difference was 0.3, corresponding to a difference in choline requirement of about 100 mg/kg (not illustrated). As there were great differences in the experimental conditions between Hansen et al.^3^ experiment and the present, these estimates should be taken as indications of magnitude of effects rather than accurate estimates.

According to our previous studies, the histological scoring of enterocyte steatosis in the pyloric caeca is among the most sensitive biomarkers of choline deficiency. As this biomarker was the most sensitive also in the present study, we decided to use it in order to obtain information on the magnitude of impact of the treatments on choline requirement^33^. The increasing rapeseed oil level from 0 to 25% increased histology score of 1.2 and 1.7 units, respectively for the small and the large fish. By employing the dose–response relationship between dietary choline level and histological steatosis score observed in Hansen et al.^3^’s study and illustrated in Fig. 7, we obtained a semi-quantitative indication of the effects of the treatments on choline requirement: for the large fish of about 650 mg/kg, for the small fish of about 450 mg/kg.

The results concerning the lipid content in the pyloric intestine mirrored the histological assessment, showing increasing lipid content with increasing rapeseed to fish oil ratio. At the same time, the lipid content was higher in the smaller fish compared to the large ones. Also, the mesenteric and hepatic lipid levels seemed to correspond to the histological scores and pyloric intestine lipid content, but with weaker statistical evidence. On the contrary, the relative weight of the pyloric intestine (OSIPI), which is a sensitive biomarker for choline deficiency in Hansen et al.^3^’s study, did not show clear effect of the rapeseed to fish oil ratio. The explanation for this apparent discrepancy may be fewer observation points and substantially larger variance in the present than in Hansen et al.^3^‘s study. The larger fish size and the exposure to the natural environmental variations may also have contributed to the overall variation in the present work.

As mentioned above, the effects of increasing ratio of rapeseed to fish oil may partly be related to the enhanced lipid digestibility, which increased lipid supply to the fish from 28.3 to about 33.0%. In our previous study^20^, a 5% increase in the level of digestible lipid in the feed increased histological scores of about 0.7 units at 8 °C, and 1.5 at 15 °C, corresponding to a shifts in the indicative choline requirement of 200 and 600 mg/kg, respectively. Based on these observations, our conclusion is therefore that the present study, in which the environmental temperature averaged 10.5 °C, indicates that the ratio of fish oil to rapeseed oil had minor effects on choline requirement and that the main cause of observed effect of lipid source on choline requirement was the supply of digestible lipid.

### Effects of fish size on the choline requirement

The effect of fish size on the indicative choline requirement was calculated by using the same dose–response relationship between dietary choline level and histological steatosis score previously published^3^. Accordingly, fish size induced a variation of 0.5 units, corresponding to a 200 mg/kg higher indicative dietary choline requirement in small compared to large fish. However, as daily feed intake and weight gain, as percent of body weight, were higher in small fish, also lipid consumption was relatively higher in small compared to large fish. At present, no experimental studies addressing effects of feed intake on steatosis symptoms and choline requirement in fish have been found. However, a life cycle field survey observing prevalence and severity of intestinal steatosis in farms along the Norwegian coast, clearly indicated that in periods with lower feed intake, i.e. when temperature is low, symptoms of steatosis are mostly absent, supposedly due to lower lipid supply and reduced demand for lipid transport capacity^34^. The present results therefore suggest that the effect of fish size, which was rather small, most likely was due to difference in lipid supply, and that effect of the differences in developmental stage, smoltification strategy, and genetic background was minor. Further studies are needed to confirm these suggestions.

### Effects on gene expression

The gene expression biomarkers observed to be most sensitive to variation in choline supply in Hansen et al.’s work^3^ comprise *plin2*, *apoA-I*, *apoA-IV* and *pcyt1α.* In particular, a clear relationship between *plin2* expression, a general marker for the lipid load of non-adipogenic cells and severity of intestinal steatosis, has been observed in several recent studies conducted in salmon^1,21,35,36^. In the present study, while the histological assessment offered a clear picture over steatosis’ severity, the gene expression results showed a less clear scenario, with the majority of the biomarker genes being unaffected by fish size or fish oil to rapeseed oil ratio. The only gene which showed an effect of fish size was *apoAIV*, significantly more expressed in larger fish. This outcome seemed to contrast with the histology results, which showed that smaller fish were the most affected by steatosis. A likely explanation for such outcome may be related to unknown factors regulating apoAIV expression. For instance, in our most recent work by Siciliani et al.^20^, in which dietary lipid level increased from 16 to 26% in choline-deficient diets (1600 g/kg), expression levels of *plin2*, *apoA-I*, *apoA-IV* and *pcyt1α* showed clear dose–response relationships with dietary fat inclusion and intestinal steatosis. However, the study also showed that interactions between the dietary lipid level and the two assessed water temperatures, 8 and 15°C, influenced the expression pattern of these genes.

Additionally, according to Hansen et al.^3^ the histologically observed degree of vacuolation and the macroscopically observed whiteness of the pyloric intestine were the indicators most closely related to steatosis. Therefore, they can be considered more reliable indicators of lipid transport mechanisms. It is also possible that, despite dietary choline deficiency was induced in the current study, the difference in lipid load among the diets was not large enough to trigger differential gene expression. In addition, the works conducted by Hansen et al.^3^ and Krogdahl et al.^2^ highlight that, even in those cases where the histology results showed a significantly high severity of lipid vacuolization, the number of differentially expressed genes was lower than expected when comparing a normal vs a steatotic morphology.

### Fatty acid metabolism

The fatty acid composition of the pyloric intestine (PI), mesenteric fat (Mes), and liver (LI), provides information which strengthens the knowledge of lipid metabolism in these tissues in relationship to dietary lipid source. The transport routes and chemical form of lipid from the intestinal lumen to the peripheral tissues in fish are not well described yet. The work of Denstadli et al.^37^, employing isotopes of 10:0 and 18:1 in a force-feeding trial with Atlantic salmon, indicates that both lipid transport route and chemical form may differ depending on the chain length of the fatty acids supplied and time after feeding. A route via the portal vein and liver is suggested for both medium and long chain fatty acids. However, a more direct route to the peripheral tissues is also indicated. The proportion of free and bound fatty acids changed with time after feeding. According to the results of the studies of effects of choline supply, it is also likely that dietary choline and lipid level, as well as feed intake, may affect lipid transport route and chemical form^1,20,2,38^. In the following discussion of fatty acid profile of the PI, Mes, and liver, it should be kept in mind that the results were obtained from fish under choline deficient conditions which may have affected the route of transport, storage, as well as lipid metabolism in the observed tissues. The fact that the fatty acids may be oxidized, elongated, integrated into phospholipids or triglycerides, and supposedly also selectively released from the tissues, makes the discussion of the results difficult. The fatty acid profile, i.e. % of total, of the Mes, overall, was closer to that of the feed, than the profile of the PI. For instance, the 14:0 level, expressed as % of total lipids, was lower in the PI fat than in the absorbed lipid, while 18:0 was higher. The 14:0 might therefore have been elongated to 18:0. Similarly, the observation that level of 20:5 was lower in PI than in the feed, whereas 22:6 was higher, indicates that 20:5 might have been converted to 22:6 in the PI. Further discussion of these aspect requires more thorough studies of fatty acid metabolism in PI and Mes.

These observations suggest that the fatty acids accumulating in the enterocytes of the PI are, to some degree, selected for storage or for passage through. Another possibility is that fatty acids stored in the enterocytes are metabolized and thereby altering the profile of the PI tissue. Recent publications, including relevant results over fatty acid metabolism in gut cell culture studies^39–43^ support this suggestion. The fact that the fatty acid profile of the Mes was more similar to that of the lipid absorbed from the feed, than to that of the PI, even if the PI precedes the Mes in the lipid transport route, may be explained by the quantities of lipid passing through the enterocytes compared to the quantity which is stored. The quantity stored in Mes was many times higher than the quantity stored in the intestine, and therefore the amounts stored in PI would not necessarily affect the lipid profile of the Mes.

The liver is commonly regarded as the key organ in lipid metabolism in an animal^44–46^. Accordingly, although correlations with level of fatty acids in the diet were observed, the liver differed the most from the levels in the absorbed lipid, supposedly reflecting substantial selectivity and high rate of metabolism of the fatty acids supplied to the organ. For instance, the relative level of 20:4n-6 and 22:6n3 was higher in the liver than in in the absorbed fat, indicating metabolic conversion from their precursors 18:2n6 and 18:3n3.

## Conclusions

The increasing dietary rapeseed oil level, and the higher feed intake of small fish induced moderate increases in severity of intestinal steatosis. The increase in rapeseed to fish oil ratio enhanced lipid digestibility and consequently lipid supply. Small fish showed relatively higher feed consumption, and consequently higher lipid intake. As increasing dietary lipid level has been shown to increase severity of steatosis, the conclusions based on the present results are that fatty acid composition and feed intake are more important than lipid source and fish size for choline requirement.

Although the overall fatty acid composition of all the observed tissues reflected that of the absorbable fat in the feed, the fatty acid composition of the mesenteric tissue resembled it more closely compared to the pyloric intestine, whereas the composition in the liver showed greater differences.

The present findings strengthen the basis for designing further experiments addressing choline requirement. A dose–response experiment with the aim to define choline requirement should be conducted using a fast-growing salmon breed, raised at a temperature on the high end of the normal range, and fed to diets containing the highest lipid level which would be used in commercial feeds.

### Supplementary Information

Supplementary Information.

## Data Availability

Raw data supporting the results presented in the paper and codes used to perform the statistical analyses are available at the following link: https://github.com/aliaksah/Effects-of-dietary-lipid-source-and-fish-size-on-steatosis-in-two-Atlantic-salmon-populations.

## References

[1] Hansen, A. K. G. *et al.* Choline supplementation prevents diet induced gut mucosa lipid accumulation in post-smolt Atlantic salmon (*Salmo salar* L.). *BMC Vet. Res.* **16**, 1–15 (2020). 32005242 10.1186/s12917-020-2252-7 PMC6995171

[2] Krogdahl, Å. *et al.* Choline and phosphatidylcholine, but not methionine, cysteine, taurine and taurocholate, eliminate excessive gut mucosal lipid accumulation in Atlantic salmon (*Salmo salar* L.). *Aquaculture* **528**, 735552 (2020). 10.1016/j.aquaculture.2020.735552

[3] Hansen, A. K. G. *et al.* Dose-response relationship between dietary choline and lipid accumulation in pyloric enterocytes of Atlantic salmon (*Salmo salar* L.) in seawater. *Br. J. Nutr.* **123**, 1081–1093 (2020). 32037990 10.1017/S0007114520000434

[4] Penn, M. Lipid malabsorption in Atlantic Salmon—The recurring problem of floating feces (2011).

[5] Zeisel, S. Choline, other methyl-donors and epigenetics. *Nutrients*. **9**(5), 445 (2017). 28468239 10.3390/nu9050445 PMC5452175

[6] NRC. Nutrient Requirements of Fish and Shrimp. *Natl. Acad. Press. Washingt* 102–134 and 186–220 (2011).

[7] Koskela, J., Pirhonen, J. & Jobling, M. Feed intake, growth rate and body composition of juvenile Baltic salmon exposed to different constant temperatures. *Aquac. Int.* **5**, 351–360 (1997). 10.1023/A:1018316224253

[8] Dhanasiri, A. K. S., Siciliani, D., Kortner, T. M. & Krogdahl, Å. Epigenetic canges in pyloric caeca of Atlantic salmon fed diets containing increasing levels of lipids and choline. *Epigenetics* **19**, (2024). 10.1080/15592294.2024.2305079 PMC10824149 38281164

[9] Li, Z. & Vance, D. E. Phosphatidylcholine and choline homeostasis. *J. Lipid Res.* **49**, 1187–1194 (2008). 18204095 10.1194/jlr.R700019-JLR200

[10] Wu, P. *et al.* Effect of dietary choline on growth, intestinal enzyme activities and relative expressions of target of rapamycin and eIF4E-binding protein2 gene in muscle, hepatopancreas and intestine of juvenile Jian carp (*Cyprinus carpio* var. Jian). *Aquaculture* **317**, 107–116 (2011). 10.1016/j.aquaculture.2011.03.042

[11] Ketola, H. G. Choline metabolism and nutritional requirement of lake trout (*Salvelinus namaycush*). *J. Anim. Sci.* **43**, 474–477 (1976). 956064 10.2527/jas1976.432474x

[12] Rumsey, G. L. Choline-betaine requirements of rainbow trout (*Oncorhynchus mykiss*). *Aquaculture* **95**, 107–116 (1991). 10.1016/0044-8486(91)90077-K

[13] Twibell, R. G. & Brown, P. B. Dietary choline requirement of juvenile yellow perch (*Perca flavescens*). *J. Nutr.* **130**, 95–99 (2000). 10613773 10.1093/jn/130.1.95

[14] Benetti, D. D. *et al.* A review on cobia, Rachycentron canadum, aquaculture. *J. World Aquac. Soc.* **52**, 691–709 (2021). 10.1111/jwas.12810

[15] Krogdahl, Å. *et al.* Effects of functional ingredients on gut inflammation in Atlantic salmon (*Salmo salar* L.). *Fish Shellfish Immunol.* **134**, 108618 (2023). 36801242 10.1016/j.fsi.2023.108618

[16] O’Shea, B., Mordue-Luntz, A. J., Fryer, R. J., Pert, C. C. & Bricknell, I. R. Determination of the surface area of a fish. *J. Fish Dis.* **29**, 437–440 (2006). 16866928 10.1111/j.1365-2761.2006.00728.x

[17] Noble, C. *et al. Welfare Indicators for farmed Atlantic salmon: tools for assessing fish welfare An FHF-financed project, led by Nofima in partnership with* (2018).

[18] Hoyle, I. *et al.* A validated macroscopic key to assess fin damage in farmed rainbow trout (*Oncorhynchus mykiss*). *Aquaculture* **270**(1–4), 142–148 (2007). 10.1016/j.aquaculture.2007.03.037

[19] Baeverfjord, G. & Krogdahl, A. Development and regression of soybean meal induced enteritis in Atlantic salmon, *Salmo salar* L., distal intestine: A comparison with the intestines of fasted fish. *J. Fish Dis.* **19**, 375–387 (1996). 10.1111/j.1365-2761.1996.tb00376.x

[20] Siciliani, D., Kortner, T. M., Berge, G. M., Hansen, A. K. & Krogdahl, Å. Effects of dietary lipid level and environmental temperature on lipid metabolism in the intestine and liver, and choline requirement in Atlantic salmon (*Salmo salar* L.) parr. *J. Nutr. Sci.* **12**, 61–62 (2023). 10.1017/jns.2023.45 PMC10214143 37252685

[21] Kortner, T. M. *et al.* Candidate reference genes for quantitative real-time PCR (qPCR) assays during development of a diet-related enteropathy in Atlantic salmon (*Salmo salar* L.) and the potential pitfalls of uncritical use of normalization software tools. *Aquaculture* **318**, 355–363 (2011). 10.1016/j.aquaculture.2011.05.038

[22] Kortner, T. M., Penn, M. H., Bjrkhem, I., Måsøval, K. & Krogdahl, Å. Bile components and lecithin supplemented to plant based diets do not diminish diet related intestinal inflammation in Atlantic salmon. *BMC Vet. Res.* (2016). 10.1186/s12917-016-0819-0 PMC5015236 27604133

[23] O’Fallon, J. V., Busboom, J. R., Nelson, M. L. & Gaskins, C. T. A direct method for fatty acid methyl ester synthesis: Application to wet meat tissues, oils, and feedstuffs. *J. Anim. Sci.* **85**, 1511–1521 (2007). 17296772 10.2527/jas.2006-491

[24] Zitzmann, S., Lüdtke, O., Robitzsch, A. & Hecht, M. On the performance of bayesian approaches in small samples: A comment on Smid, McNeish, Miocevic, and van de Schoot (2020). *Struct. Equ. Model.* **28**, 40–50 (2021). 10.1080/10705511.2020.1752216

[25] Neill, J. W. & Johnson, D. E. Testing linear regression function adequacy without replication. *Ann. Stat.* (2007).

[26] Hoeting, J. A., Madigan, D., Raftery, A. E. & Volinsky, C. T. Bayesian model averaging: A tutorial. *Stat. Sci.* **14**, 382–401 (1999).

[27] Virgilio, G.-R. *Bayesian inference with INLA* (CRC Press, 2020).

[28] Liu, A., Mazumder, D., Pirozzi, I., Sammut, J. & Booth, M. The effect of dietary choline and water temperature on the contribution of raw materials to the muscle tissue of juvenile yellowtail kingfish (*Seriola lalandi*): An investigation using a stable isotope mixing model. *Anim. Feed Sci. Technol.* **280**, 115087 (2021). 10.1016/j.anifeedsci.2021.115087

[29] Kass, R. E. & Raftery, A. E. Bayes factors and model uncertainty. *J. Am. Stat. Assoc.* **90**, 773 (1995). 10.1080/01621459.1995.10476572

[30] Berger, V. W. & Zhou, Y. Kolmogorov-Smirnov test: Overview. *Wiley Stat. Ref. Stat. Ref. Online* (2014).

[31] Karalazos, V., Bendiksen, E. Å. & Bell, J. G. Interactive effects of dietary protein/lipid level and oil source on growth, feed utilisation and nutrient and fatty acid digestibility of Atlantic salmon. *Aquaculture* **311**, 193–200 (2011). 10.1016/j.aquaculture.2010.11.022

[32] Ng, W. K., Sigholt, T. & Bell, J. G. The influence of environmental temperature on the apparent nutrient and fatty acid digestibility in Atlantic salmon (*Salmo salar* L.) fed finishing diets containing different blends of fish oil, rapeseed oil and palm oil. *Aquac. Res.* **35**, 1228–1237 (2004). 10.1111/j.1365-2109.2004.01131.x

[33] Krogdahl, Å. *et al.* Dietary fish meal level and a package of choline, β -glucan, and nucleotides modulate gut function, microbiota, and health in Atlantic salmon (*Salmo salar*, L.). *Aquac. Nutr.* **2023**, 1–29 (2023). 10.1155/2023/5422035 PMC9973201 36860972

[34] Krogdahl, Å. *et al.* GutMatters—Defining and improving intestinal health in farmed salmon. (2022).

[35] Wang, J. *et al.* Gut immune functions and health in Atlantic salmon (*Salmo salar*) from late freshwater stage until one year in seawater and effects of functional ingredients: A case study from a commercial sized research site in the Arctic region. *Fish Shellfish Immunol.* **106**, 1106–1119 (2020). 32941976 10.1016/j.fsi.2020.09.019

[36] Wang, J. *et al.* Gut Health and microbiota in out-of-season Atlantic salmon (*Salmo salar* L.) smolts before and after seawater transfer under commercial arctic conditions: Modulation by functional feed ingredients. *Mar. Sci. Front.* (2022).

[37] Denstadli, V. *et al.* Medium-chain and long-chain fatty acids have different postabsorptive fates in Atlantic salmon. *J. Nutr.* **141**, 1618–1625 (2011). 21753060 10.3945/jn.111.141820

[38] Li, Y. *et al.* Gut health and vaccination response in pre-smolt Atlantic salmon (*Salmo salar *) fed black soldier fly (*Hermetia illucens *) larvae meal. *Fish Shellfish Immunol.* **86**, 1106–1113 (2019). 30590165 10.1016/j.fsi.2018.12.057

[39] Mellery, J. *et al.* Temperature increase negatively affects the fatty acid bioconversion capacity of rainbow trout (*Oncorhynchus mykiss*) fed a linseed oil-based diet. *PLoS One* **11**, 1–24 (2016). 10.1371/journal.pone.0164478 PMC5063364 27736913

[40] Tocher, D. R. Metabolism and functions of lipids and fatty acids in teleost fish. *Rev. Fish. Sci.* **11**, 107–184 (2003). 10.1080/713610925

[41] Jin, Y. *et al.* A systemic study of lipid metabolism regulation in salmon fingerlings and early juveniles fed plant oil. *Br. J. Nutr.* (2018). 10.1017/S0007114518001885 30064538

[42] Selvam, C., Saito, T., Sissener, N. H., Philip, A. J. P. & Sæle, Ø. Intracellular trafficking of fatty acids in the fish intestinal epithelial cell line RTgutGC. *Front. Mar. Sci.* **9**, 1–15 (2022).35450130 10.3389/fmars.2022.954773

[43] Jin, Y. *et al.* Diet and life stage-associated lipidome remodeling in Atlantic salmon. *J. Agric. Food Chem.* **69**, 3787–3796 (2021). 33754702 10.1021/acs.jafc.0c07281 PMC8041299

[44] Nguyen, P. *et al.* Liver lipid metabolism. *J. Anim. Physiol. Anim. Nutr. (Berl)* **92**, 272–283 (2008). 18477307 10.1111/j.1439-0396.2007.00752.x

[45] Baez, R. V. *Lipid metabolism* (InTech, 2013).

[46] Hardy, R. W. & Kaushik, S. J. Fish nutrition. *Fish Nutr.* (2022).

