# Supplementary material for: Effects of dietary fish to rapeseed oil ratio on steatosis symptoms in Atlantic salmon (Salmo salar L) of different sizes
Source: Sci Rep. 2024 Aug 3;14:18006. doi: 10.1038/s41598-024-68434-3 (PMC11297975; doi:10.1038/s41598-024-68434-3)
Supplement: Supplementary file 1 — Supplementary Information. [file 41598_2024_68434_MOESM1_ESM.docx]

***Table S1.*** *Primer pair sequences, efficiency, amplicon size and annealing temperature for the genes used for real-time PCR.*

**Gene name**

**Gene symbol**

**Forward primer**

**Reverse primer**

**Amplicon**

**size**

**Annealing**

**temperature**

**Efficiency**

**Acc. number**

***Target genes***

Perilipin2

*plin2*

CCCAGGTCTACTCCAGCTTC

CAGCGACTCCTTCATCTTGC

104

60

2.0

BT072598

Apolipoprotein A

-

I

*apoA*

*-*

*I*

CTGGTCCTCGCACTAACCAT

TGGACCTCTGTGCAGTCAAC

144

60

2.0

NM_001123663

Apolipoprotein A

-

IV

*apoA*

*-*

*IV*

CAGGACCAGTCTCAGCAACA

GTTGACTTCCTGTGCCACCT

131

60

1.9

BT048822

Choline

-

phosphate cytidylyltransferase

*pcyt1a*

CGGGTCTATGCAGATGGAAT

GCTCGTCCTCGTTCATCACT

166

60

2.1

BT045986

***Reference genes***

| RNA polymerase II | *rnapoll* | CCAATACATGACCAAATATGAAAGG | ATGATGATGGGGATCTTCCTGC | 157 | 60 | 2.0 | BG936649 |
| --- | --- | --- | --- | --- | --- | --- | --- |
| hypoxanthine phosphoribosyl transferase 1 | *hprt1* | CCGCCTCAAGAGCTACTGTAAT | GTCTGGAACCTCAAACCCTATG | 255 | 60 | 1.9 | BT043501 |
| glyceraldehyde-3-phosphate dehydrogenase | *gapdh* | AAGTGAAGCAGGAGGGTGGAA | CAGCCTCACCCCATTTGATG | 96 | 60 | 2.1 | BT050045 |

***Table S2.*** *Explanation of terms and abbreviations used for the Bayesian statistics.*

|  | Explanations |
| --- | --- |
| Best model | Model with the highest posterior probability |
| Prob | Posterior probability of the best model |
| BF12 | Bayes factor between the best model and the model with the second highest posterior probability |
| Model efficiency | Values of BF12 indicating evidence for the selected models |
| Negl | Value indicating negligible evidence for the selected model (BF12 between 1 and 3.2) |
| Subst | Value indicating substantial evidence for the selected model (BF12 between 3.2 and 10) |
| Strong | Value indicating strong evidence for the selected model (BF12 between 10 and 100) |
| Decis | Value indicating decisive evidence for the selected model (BF12 >100) |
| B0 | Posterior mode of the intercept |
| B0L | Lower bound of 95% credible interval for the intercept |
| B0U | Upper bound of 95% credible interval for the intercept |
| BS | Posterior mode of the effect for Size |
| BSL | Lower bound of 95% credible interval for the effect for Size |
| BSU | Upper bound of 95% credible interval for the effect for Size |
| BR | Posterior mode of the effect for Rape Oil |
| BRL | Lower bound of 95% credible interval for the effect for Rape Oil |
| BRU | Upper bound of 95% credible interval for the effect for Rape Oil |
| BRS0 | Posterior mode of the effect for Rape Oil for small fish |
| BRS0L | Lower bound of 95% credible interval for the effect for Rape Oil for small fish |
| BRS0U | Upper bound of 95% credible interval for the effect for Rape Oil for small fish |
| BRS1 | Posterior mode of the effect for Rape Oil for large fish |
| BRS1L | Lower bound of 95% credible interval for the effect for Rape Oil for large fish |
| BRS1U | Upper bound of 95% credible interval for the effect for Rape Oil for large fish |
| KS | P-value for the Kolmogorov – Smirnov test for the residuals |

***Table S3.*** *Results of statistical evaluation of effects of fish size and rapeseed oil level in the feed on final weight (BW), conditions factor (CF), growth (TGC), yield, organosomatic indices of the*

*pyloric organ package (OSIPI1) and pyloric intestine cleaned of fatty tissue (OSIPI2), mid intestine (MISI), and distal intestine (DISI)*.*

|  | Best model | Prob | BF12 | Model efficiency | B0 | B0L | B0U | BS | BSL | BSU | KS |
| --- | --- | --- | --- | --- | --- | --- | --- | --- | --- | --- | --- |
| BW | m3 | 1.000 | 11465 | Decis | 8.75 | 8.72 | 8.78 | -0.72 | -0.76 | -0.67 | 0 |
| TGC | m3 | 0.999 | 919 | Decis | 1.37 | 1.33 | 1.41 | 0.187 | 0.13 | 0.244 | 0 |
| FCR | m1 | 1.000 | 5157 | Decis | 1.12 | 1.08 | 1.17 | 0 | 0 | 0 | 0.05 |
| CF | m1 | 0.999 | 2408 | Decis | 0.92 | 0.91 | 0.93 | 0 | 0 | 0 | 0 |
| OSIPI1 | m1 | 0.995 | 208 | Decis | 2.02 | 2.00 | 2.04 | 0 | 0 | 0 | 0.05 |
| OSIPI2 | m3 | 0.978 | 45 | Strong | 1.16 | 1.13 | 1.18 | 0.158 | 0.12 | 0.195 | 0.3 |
| MISI | m1 | 0.999 | 898 | Decis | 0.19 | 0.18 | 0.19 | 0 | 0 | 0 | 0 |
| DISI | m1 | 0.921 | 12 | Strong | 0.4 | 0.38 | 0.41 | 0 | 0 | 0 | 0 |
| LISI | m1 | 0.999 | 2161 | Decis | 0.82 | 0.81 | 0.83 | 0 | 0 | 0 | 0 |

- *Explanations to the abbreviations are given in Table S2.*

***Table S4.*** *Results of statistical evaluation of digestibility of crude protein, sum of fatty acids (FA) and fatty acid**

|  | Best model | Prob | BF12 | Model efficiency | B0 | B0L | B0U | BS | BSL | BSU | KS |
| --- | --- | --- | --- | --- | --- | --- | --- | --- | --- | --- | --- |
| Crude protein | m3 | 0.863 | 6 | Subst | 88.1 | 87.3 | 89.0 | -2.41 | -3.63 | -1.19 | 0.811 |
| Sum FA | m2 | 0.961 | 25 | Strong | 80.2 | 78.4 | 82.0 | 0.62 | 0.49 | 0.75 | 0.210 |
| 12:0 | m1 | 0.857 | 9 | Subst | 91.3 | 89.7 | 93.0 | 0 | 0 | 0 | 0.110 |
| 14:0 | m2 | 0.944 | 18 | Strong | 72.8 | 69.9 | 75.7 | 0.74 | 0.54 | 0.95 | 0.988 |
| 15:0 | m2 | 0.935 | 15 | Strong | 66.2 | 62.9 | 69.5 | 0.87 | 0.64 | 1.10 | 0.996 |
| 16:0 | m2 | 0.922 | 12 | Strong | 57.0 | 53.2 | 60.8 | 1.14 | 0.87 | 1.40 | 0.997 |
| 17:0 | m2 | 0.916 | 11 | Strong | 54.3 | 49.9 | 58.8 | 1.30 | 0.99 | 1.62 | 0.989 |
| 18:0 | m2 | 0.886 | 10 | Strong | 36.9 | 31.8 | 42.0 | 1.03 | 0.67 | 1.39 | 0.928 |
| 20:0 | m2 | 0.844 | 10 | Strong | 49.3 | 45.0 | 53.5 | 0.77 | 0.47 | 1.07 | 0.812 |
| 22:0 | m1 | 0.421 | 1 | Negl | -1.0 | -8.9 | 6.9 | 0 | 0 | 0 | 0.300 |
| 24:0 | m2 | 0.862 | 7 | Subst | 38.8 | 33.0 | 44.7 | 1.21 | 0.80 | 1.62 | 0.967 |
| 16:1n7 | m1 | 0.901 | 12 | Strong | 97.4 | 96.5 | 98.4 | 0 | 0 | 0 | 0.000 |
| 18:1n9c | m1 | 0.473 | 1 | Negl | 96.1 | 94.7 | 97.5 | 0 | 0 | 0 | 0.001 |
| 20:1 | m2 | 0.530 | 1 | Negl | 88.9 | 86.7 | 91.1 | 0.32 | 0.17 | 0.48 | 0.038 |
| 22:1n9 | m2 | 0.929 | 14 | Strong | 80.2 | 77.7 | 82.7 | 0.71 | 0.53 | 0.88 | 0.131 |
| 22:1n11 | m1 | 0.583 | 2 | Negl | 91.6 | 89.6 | 93.5 | 0 | 0 | 0 | 0.001 |
| 24:1 | m2 | 0.933 | 15 | Strong | 68.4 | 65.2 | 71.7 | 0.83 | 0.60 | 1.05 | 0.249 |
| 18:2n6c | m2 | 0.893 | 12 | Strong | 90.9 | 89.3 | 92.5 | 0.32 | 0.20 | 0.43 | 0.811 |
| 18:3n3 | m2 | 0.893 | 14 | Strong | 92.2 | 90.5 | 93.8 | 0.32 | 0.21 | 0.44 | 0.759 |
| 18:3n6 | m1 | 1.000 | 20799 | Decis | 100 | 100 | 100 | 0 | 0 | 0 | 0.000 |
| 20:2 | m1 | 0.863 | 6 | Subst | 97.5 | 96.8 | 98.1 | 0 | 0 | 0 | 0.000 |
| 20:3n3 | m1 | 0.756 | 5 | Subst | 42.9 | 37.4 | 48.5 | 0 | 0 | 0 | 0.454 |
| 20:3n6 | m1 | 0.858 | 6 | Subst | 95.8 | 94.5 | 97.1 | 0 | 0 | 0 | 0.000 |
| 20:4n6 | m1 | 0.902 | 9 | Subst | 97.7 | 97.1 | 98.4 | 0 | 0 | 0 | 0.000 |
| 20:5n3 | m1 | 0.944 | 19 | Strong | 98.5 | 98.0 | 99.1 | 0 | 0 | 0 | 0.000 |
| 22:5n3 | m1 | 0.804 | 4 | Subst | 96.8 | 96.1 | 97.6 | 0 | 0 | 0 | 0.000 |
| 22:6n3 | m1 | 0.928 | 13 | Strong | 96.4 | 95.8 | 97.0 | 0 | 0 | 0 | 0.000 |

- *Explanations to the abbreviations are given in Table S2.*

***Table S5.*** *Results of statistical evaluation of effects of fish size and rapeseed oil level in the feed on plasma biomarkers**

|  | Model | Prob | BF12 | Model efficiency | B0 | B0L | B0U | BS | BSL | BSU | KS |
| --- | --- | --- | --- | --- | --- | --- | --- | --- | --- | --- | --- |
| TG | m1 | 0.957 | 22 | Strong | 2.7 | 2.6 | 2.8 | 0 | 0 | 0 | 0 |
| FFS | m1 | 0.686 | 2 | Negl | 0.32 | 0.29 | 0.34 | 0 | 0 | 0 | 0 |
| Chol | m1 | 0.992 | 128 | Decis | 7.5 | 7.3 | 7.7 | 0 | 0 | 0 | 0 |
| Glu | m1 | 0.920 | 12 | Strong | 6.8 | 6.6 | 7.0 | 0 | 0 | 0 | 0 |
| ALT | m3 | 0.995 | 228 | Decis | 17.3 | 15.3 | 19.4 | 7.4 | 4.5 | 10.3 | 0 |

- *Explanations to the abbreviations are given in Table S1. ALT=Alanine transferase, FFS: free fatty acids, Glu=glucose, Chol=Cholesterol, TG=triglycerides.*

***Table S6.*** *Results of statistical evaluation of effects of fish size and rapeseed oil level in the feed on expression of genes selected for responsibility to level of supply of choline.*

|  | Best model | Prob | BF12 | Model  Efficiency | B0 | B0L | B0U | BS | BSL | BSU | KS |
| --- | --- | --- | --- | --- | --- | --- | --- | --- | --- | --- | --- |
| apoA-I | m1 | 0.954 | 22 | Strong | 18.9 | 17.7 | 20.1 | 0 | 0 | 0 | 0.383 |
| apoA-IV | m3 | 0.965 | 32 | Strong | 41.7 | 38.9 | 44.4 | -8.1 | -11.9 | -4.2 | 0.832 |
| pcyt1a | m1 | 0.997 | 294 | Decis | 0.099 | 0.092 | 0.106 | 0 | 0 | 0 | 0.628 |
| adph | m1 | 0.844 | 8 | Subst | 7.42 | 6.675 | 8.162 | 0 | 0 | 0 | 0.436 |

- *Explanations to the abbreviations are given in Table S2.*

***Table S7a.*** *Results of evaluation of effects of fish size and rapeseed oil level in the feed on fatty acid content in absorbed fat (Feed), mesenteric fatty tissue (Mes), pyloric tissue (PI) and liver (LI) expressed as area % of sum of fatty acids**

|  | Best model | Prob | BF12 | B0 | B0L | B0U | BS | BSL | BSU | BR | BRL | BRU | BRS0 | BRS0L | BRS0U | BRS1 | BRS1L | BRS1U | KS |  |
| --- | --- | --- | --- | --- | --- | --- | --- | --- | --- | --- | --- | --- | --- | --- | --- | --- | --- | --- | --- | --- |
| 12:0 Feed | m2 | 1.000 | 13826 | 0.118 | 0.114 | 0.122 | 0 | 0 | 0 | -0.004 | -0.004 | -0.003 | 0 | 0 | 0 | 0 | 0 | 0 | 0.764 |  |
| 14:0 Feed | m2 | 0.996 | 221 | 7.39 | 7.14 | 7.63 | 0 | 0 | 0 | -0.228 | -0.245 | -0.211 | 0 | 0 | 0 | 0 | 0 | 0 | 0.687 |  |
| 16:0 Feed | m2 | 0.988 | 85 | 16.4 | 15.8 | 17.0 | 0 | 0 | 0 | -0.311 | -0.355 | -0.267 | 0 | 0 | 0 | 0 | 0 | 0 | 0.985 |  |
| 18:0 Feed | m1 | 0.992 | 234 | 2.40 | 2.27 | 2.54 | 0 | 0 | 0 | 0 | 0 | 0 | 0 | 0 | 0 | 0 | 0 | 0 | 0.048 |  |
| 20:0 Feed | m1 | 0.736 | 3 | 0.619 | 0.586 | 0.652 | 0 | 0 | 0 | 0 | 0 | 0 | 0 | 0 | 0 | 0 | 0 | 0 | 0.125 |  |
| 16:1n7 Feed | m2 | 0.994 | 169 | 9.65 | 9.32 | 9.98 | 0 | 0 | 0 | -0.321 | -0.345 | -0.298 | 0 | 0 | 0 | 0 | 0 | 0 | 0.182 |  |
| 18:1n9c Feed | m2 | 0.973 | 36 | 17.6 | 16.1 | 19.0 | 0 | 0 | 0 | 1.296 | 1.191 | 1.4 | 0 | 0 | 0 | 0 | 0 | 0 | 0.053 |  |
| 20:1 Feed | m2 | 0.998 | 720 | 2.03 | 1.99 | 2.07 | 0 | 0 | 0 | -0.015 | -0.017 | -0.012 | 0 | 0 | 0 | 0 | 0 | 0 | 0.501 |  |
| 18:2n6c Feed | m2 | 0.985 | 68 | 6.20 | 5.37 | 7.03 | 0 | 0 | 0 | 0.541 | 0.483 | 0.599 | 0 | 0 | 0 | 0 | 0 | 0 | 0.063 |  |
| 18:3n3 Feed | m2 | 0.996 | 236 | 1.42 | 1.19 | 1.66 | 0 | 0 | 0 | 0.217 | 0.2 | 0.233 | 0 | 0 | 0 | 0 | 0 | 0 | 0.036 |  |
| 20:3n3 Feed | m1 | 0.582 | 1 | 0.023 | 0.015 | 0.031 | 0 | 0 | 0 | 0 | 0 | 0 | 0 | 0 | 0 | 0 | 0 | 0 | 0.226 |  |
| 20:4n6 Feed | m2 | 0.999 | 965 | 1.49 | 1.43 | 1.55 | 0 | 0 | 0 | -0.052 | -0.056 | -0.048 | 0 | 0 | 0 | 0 | 0 | 0 | 0.550 |  |
| 20:5n3 Feed | m2 | 0.986 | 70 | 19.4 | 18.6 | 20.2 | 0 | 0 | 0 | -0.663 | -0.719 | -0.608 | 0 | 0 | 0 | 0 | 0 | 0 | 0.402 |  |
| 22:5n3 Feed | m2 | 0.998 | 526 | 2.22 | 2.12 | 2.33 | 0 | 0 | 0 | -0.077 | -0.084 | -0.07 | 0 | 0 | 0 | 0 | 0 | 0 | 0.989 |  |
| 22:6n3 Feed | m2 | 0.994 | 156 | 8.19 | 7.84 | 8.55 | 0 | 0 | 0 | -0.266 | -0.291 | -0.241 | 0 | 0 | 0 | 0 | 0 | 0 | 0.761 |  |
| 12:0 Mes | m1 | 0.997 | 360 | 0.038 | 0.033 | 0.044 | 0 | 0 | 0 | 0 | 0 | 0 | 0 | 0 | 0 | 0 | 0 | 0 | 0.028 |  |
| 14:0 Mes | m2 | 0.996 | 279 | 5.71 | 5.54 | 5.87 | 0 | 0 | 0 | -0.165 | -0.176 | -0.153 | 0 | 0 | 0 | 0 | 0 | 0 | 0.999 |  |
| 16:0 Mes | m2 | 0.503 | 1 | 16.4 | 16.1 | 16.8 | 0 | 0 | 0 | -0.331 | -0.358 | -0.304 | 0 | 0 | 0 | 0 | 0 | 0 | 0.758 |  |
| 18:0 Mes | | m2 | 0.771 | 7 | 5.03 | 4.70 | 5.36 | 0 | 0 | 0 | -0.072 | -0.095 | -0.048 | 0 | 0 | 0 | 0 | 0 | 0 | 0.793 |
| 20:0 Mes | m1 | 0.999 | 808 | 0.466 | 0.437 | 0.495 | 0 | 0 | 0 | 0 | 0 | 0 | 0 | 0 | 0 | 0 | 0 | 0 | 0.045 |  |
| 16:1n7 Mes | m2 | 0.988 | 85 | 6.85 | 6.52 | 7.18 | 0 | 0 | 0 | -0.228 | -0.251 | -0.205 | 0 | 0 | 0 | 0 | 0 | 0 | 0.990 |  |
| 18:1n9c Mes | m2 | 0.938 | 15 | 14.4 | 13.2 | 15.5 | 0 | 0 | 0 | 1.202 | 1.121 | 1.282 | 0 | 0 | 0 | 0 | 0 | 0 | 0.989 |  |
| 20:1 Mes | m2 | 0.983 | 65 | 1.27 | 1.11 | 1.42 | 0 | 0 | 0 | 0.053 | 0.042 | 0.064 | 0 | 0 | 0 | 0 | 0 | 0 | 0.148 |  |
| 18:2n6c Mes | m2 | 0.961 | 25 | 3.18 | 2.76 | 3.60 | 0 | 0 | 0 | 0.448 | 0.418 | 0.477 | 0 | 0 | 0 | 0 | 0 | 0 | 0.767 |  |
|  |  |  |  |  |  |  |  |  |  |  |  |  |  |  |  |  |  |  |  |  |
| 18:3n3 Mes | m2 | 0.996 | 280 | 1.39 | 1.21 | 1.57 | 0 | 0 | 0 | 0.129 | 0.117 | 0.142 | 0 | 0 | 0 | 0 | 0 | 0 | 0.875 |  |
| 20:3n3 Mes | m2 | 1.000 | 2393 | 0.196 | 0.177 | 0.215 | 0 | 0 | 0 | 0.013 | 0.012 | 0.015 | 0 | 0 | 0 | 0 | 0 | 0 | 0.303 |  |
| 20:4n6 Mes | m2 | 0.962 | 26 | 1.67 | 1.55 | 1.78 | 0 | 0 | 0 | -0.045 | -0.053 | -0.037 | 0 | 0 | 0 | 0 | 0 | 0 | 0.326 |  |
| 20:5n3 Mes | m2 | 0.989 | 94 | 10.9 | 10.3 | 11.5 | 0 | 0 | 0 | -0.359 | -0.4 | -0.317 | 0 | 0 | 0 | 0 | 0 | 0 | 0.257 |  |
| 22:5n3 Mes | m2 | 0.972 | 35 | 2.89 | 2.73 | 3.05 | 0 | 0 | 0 | -0.084 | -0.095 | -0.073 | 0 | 0 | 0 | 0 | 0 | 0 | 0.385 |  |
| 22:6n3 Mes | m2 | 0.955 | 22 | 8.49 | 7.88 | 9.11 | 0 | 0 | 0 | -0.216 | -0.259 | -0.173 | 0 | 0 | 0 | 0 | 0 | 0 | 0.406 |  |
| 12:0 PI | m1 | 0.996 | 311 | 0.037 | 0.03 | 0.043 | 0 | 0 | 0 | 0 | 0 | 0 | 0 | 0 | 0 | 0 | 0 | 0 | 0.078 |  |
| 14:0 PI | m5 | 0.986 | 80 | 4.39 | 4.32 | 4.46 | -0.89 | -0.99 | -0.79 | 0 | 0 | 0 | -0.04 | -35.9 | 35.8 | -0.01 | -35.8 | 35.8 | 0.708 |  |
| 16:0 PI | m4 | 0.849 | 6 | 12.1 | 11.8 | 12.5 | -0.98 | -1.31 | -0.64 | -0.142 | -0.163 | -0.121 | 0 | 0 | 0 | 0 | 0 | 0 | 0.995 |  |
| 18:0 PI | m2 | 0.827 | 6 | 3.00 | 2.93 | 3.07 | 0 | 0 | 0 | -0.018 | -0.023 | -0.013 | 0 | 0 | 0 | 0 | 0 | 0 | 0.914 |  |
| 20:0 PI | m1 | 0.923 | 12 | 0.337 | 0.325 | 0.35 | 0 | 0 | 0 | 0 | 0 | 0 | 0 | 0 | 0 | 0 | 0 | 0 | 0.056 |  |
| 16:1n7 PI | m5 | 0.972 | 60 | 5.66 | 5.54 | 5.78 | -1.26 | -1.43 | -1.08 | 0 | 0 | 0 | -0.07 | -35.9 | 35.7 | -0.01 | -35.8 | 35.8 | 0.082 |  |
| 18:1n9c PI | m5 | 0.975 | 48 | 24.2 | 23.4 | 25.1 | 6.833 | 5.624 | 8.039 | 0 | 0 | 0 | 0.378 | -35.4 | 36.2 | 0.06 | -35.7 | 35.9 | 0.882 |  |
| 20:1 PI | m1 | 0.903 | 11 | 2.85 | 2.76 | 2.95 | 0 | 0 | 0 | 0 | 0 | 0 | 0 | 0 | 0 | 0 | 0 | 0 | 0.545 |  |
| 18:2n6c PI | m5 | 0.881 | 13 | 8.89 | 8.60 | 9.17 | 2.206 | 1.802 | 2.609 | 0 | 0 | 0 | 0.127 | -35.7 | 35.9 | 0.02 | -35.8 | 35.8 | 1.000 |  |
| 18:3n3 PI | m4 | 0.726 | 3 | 3.58 | 3.33 | 3.82 | 0.978 | 0.738 | 1.22 | 0.079 | 0.064 | 0.094 | 0 | 0 | 0 | 0 | 0 | 0 | 0.349 |  |
| 20:3n3 PI | m1 | 0.589 | 2 | 0.454 | 0.407 | 0.502 | 0 | 0 | 0 | 0 | 0 | 0 | 0 | 0 | 0 | 0 | 0 | 0 | 0.383 |  |
| 20:4n6 PI | m2 | 0.700 | 5 | 0.594 | 0.525 | 0.662 | 0 | 0 | 0 | -0.017 | -0.022 | -0.012 | 0 | 0 | 0 | 0 | 0 | 0 | 0.856 |  |
| 20:5n3 PI | m5 | 0.835 | 7 | 8.56 | 8.24 | 8.87 | -2.421 | -2.865 | -1.98 | 0 | 0 | 0 | -0.124 | -35.9 | 35.7 | -0.02 | -35.8 | 35.8 | 0.246 |  |
| 22:5n3 PI | m5 | 0.951 | 35 | 3.44 | 3.37 | 3.50 | -0.73 | -0.828 | -0.63 | 0 | 0 | 0 | -0.044 | -35.9 | 35.8 | -0.01 | -35.8 | 35.8 | 0.850 |  |
| 22:6n3 PI | m2 | 0.520 | 1 | 5.29 | 4.92 | 5.67 | 0 | 0 | 0 | -0.12 | -0.146 | -0.093 | 0 | 0 | 0 | 0 | 0 | 0 | 0.913 |  |
| 12:0 LI | m1 | 1 | 18612 | 0.0 | 0.0 | 0.0 | 0 | 0 | 0 | 0 | 0 | 0 | 0 | 0 | 0 | 0 | 0 | 0 | 0 |  |
| 14:0 LI | m2 | 0.811 | 4.4 | 1.8 | 1.7 | 2.0 | 0 | 0 | 0 | -0.043 | -0.052 | -0.035 | 0 | 0 | 0 | 0 | 0 | 0 | 0.933 |  |
| 16:0 LI | m2 | 0.889 | 9.0 | 13.5 | 12.7 | 14.2 | 0 | 0 | 0 | -0.192 | -0.245 | -0.138 | 0 | 0 | 0 | 0 | 0 | 0 | 0.219 |  |
| 18:0 LI | m2 | 0.639 | 1.8 | 8.5 | 8.0 | 9.0 | 0 | 0 | 0 | -0.128 | -0.16 | -0.095 | 0 | 0 | 0 | 0 | 0 | 0 | 0.686 |  |
| 20:0 LI | m1 | 0.998 | 534 | 0.3 | 0.3 | 0.3 | 0 | 0 | 0 | 0 | 0 | 0 | 0 | 0 | 0 | 0 | 0 | 0 | 0.377 |  |
| 16:1n7 LI | m2 | 0.516 | 1.3 | 2.7 | 2.4 | 3.0 | 0 | 0 | 0 | -0.066 | -0.087 | -0.044 | 0 | 0 | 0 | 0 | 0 | 0 | 0.378 |  |
| 18:1n9c LI | m2 | 0.815 | 4.7 | 10.4 | 8.1 | 12.6 | 0 | 0 | 0 | 0.86 | 0.701 | 1.02 | 0 | 0 | 0 | 0 | 0 | 0 | 0.392 |  |
| 20:1 LI | m2 | 0.994 | 162 | 1.1 | 0.9 | 1.3 | 0 | 0 | 0 | 0.112 | 0.097 | 0.128 | 0 | 0 | 0 | 0 | 0 | 0 | 0.46 |  |
| 18:2n6c LI | m2 | 0.964 | 27 | 2.1 | 1.5 | 2.7 | 0 | 0 | 0 | 0.303 | 0.262 | 0.343 | 0 | 0 | 0 | 0 | 0 | 0 | 0.352 |  |
| 18:3n3 LI | m2 | 0.99 | 107 | 0.7 | 0.5 | 0.9 | 0 | 0 | 0 | 0.078 | 0.063 | 0.093 | 0 | 0 | 0 | 0 | 0 | 0 | 0.283 |  |
| 20:3n3 LI | m1 | 0.994 | 191 | 0.5 | 0.4 | 0.6 | 0 | 0 | 0 | 0 | 0 | 0 | 0 | 0 | 0 | 0 | 0 | 0 | 0.682 |  |
| 20:4n6 LI | m2 | 0.822 | 4.9 | 4.6 | 4.3 | 4.9 | 0 | 0 | 0 | -0.092 | -0.116 | -0.069 | 0 | 0 | 0 | 0 | 0 | 0 | 0.046 |  |
| 20:5n3 LI | m2 | 0.965 | 27.6 | 14.2 | 13.5 | 14.9 | 0 | 0 | 0 | -0.317 | -0.365 | -0.269 | 0 | 0 | 0 | 0 | 0 | 0 | 0.337 |  |
| 22:5n3 LI | m2 | 0.637 | 1.8 | 6.1 | 5.9 | 6.4 | 0 | 0 | 0 | -0.166 | -0.184 | -0.148 | 0 | 0 | 0 | 0 | 0 | 0 | 0.681 |  |
| 22:6n3 LI | m2 | 0.862 | 11.8 | 18.6 | 17.3 | 19.9 | 0 | 0 | 0 | -0.265 | -0.358 | -0.173 | 0 | 0 | 0 | 0 | 0 | 0 |  |  |

**Explanations to the abbreviations are given in Table 2*

***Table S7b.*** *Results of evaluation of effects of fish size and rapeseed oil level in the feed on fatty acid level in absorbed fat (Feed), mesenteric fatty tissue (Mes), pyloric tissue (PI) and liver (LI) expressed as g/kg feed or tissue.*

Model

Best Prob BF12 Efficiency B0 B0L B0U BS BSL BSU BR BRL BRU KS

| 12:0 Feed | m2 | 0.999 | 2915 | Decis | 0.22 | 0.21 | 0.23 | 0 | 0 | 0 | -0.005 | -0.006 | -0.005 | 0.251 |
| --- | --- | --- | --- | --- | --- | --- | --- | --- | --- | --- | --- | --- | --- | --- |
| 14:0 Feed | m2 | 0.990 | 104 | Decis | 13.8 | 13.2 | 14.3 | 0 | 0 | 0 | -0.35 | -0.39 | -0.32 | 0.994 |
| 16:0 Feed | m2 | 0.809 | 5 | Subst | 30.3 | 28.5 | 32.0 | 0 | 0 | 0 | -0.31 | -0.43 | -0.19 | 0.967 |
| 18:0 Feed | m1 | 0.987 | 89 | Strong | 5.11 | 4.78 | 5.45 | 0 | 0 | 0 | 0 | 0 | 0 | 0.248 |
| 20:0 Feed | m1 | 0.997 | 436 | Decis | 1.3 | 1.25 | 1.37 | 0 | 0 | 0 | 0 | 0 | 0 | 0.319 |
| 16:1n7 Feed | m2 | 0.989 | 92 | Strong | 17.9 | 17.5 | 18.3 | 0 | 0 | 0 | -0.51 | -0.54 | -0.48 | 0.984 |
| 18:1n9c Feed | m2 | 0.965 | 27 | Strong | 28.6 | 27.5 | 29.8 | 0 | 0 | 0 | 3.73 | 3.65 | 3.81 | 0.354 |
| 20:1 Feed | m1 | 0.606 | 2 | Negl | 3.9 | 3.81 | 4.07 | 0 | 0 | 0 | 0 | 0 | 0 | 0.145 |
| 18:2n6c Feed | m2 | 0.990 | 97 | Strong | 10.0 | 9.43 | 10.55 | 0 | 0 | 0 | 1.52 | 1.48 | 1.56 | 0.029 |
| 18:3n3 Feed | m2 | 0.997 | 331 | Decis | 2.08 | 1.94 | 2.22 | 0 | 0 | 0 | 0.58 | 0.57 | 0.59 | 0.687 |
| 20:3n3 Feed | m1 | 0.780 | 4 | Subst | 0.05 | 0.03 | 0.06 | 0 | 0 | 0 | 0 | 0 | 0 | 0.395 |
| 20:4n6 Feed | m2 | 0.999 | 680 | Decis | 2.8 | 2.71 | 2.83 | 0 | 0 | 0 | -0.08 | -0.09 | -0.08 | 0.126 |
| 20:5n3 Feed | m2 | 0.982 | 56 | Strong | 36.1 | 35.2 | 37.0 | 0 | 0 | 0 | -1.06 | -1.13 | -1.00 | 0.093 |
| 22:5n3 Feed | m2 | 0.997 | 336 | Decis | 4.1 | 4.00 | 4.26 | 0 | 0 | 0 | -0.12 | -0.13 | -0.12 | 0.396 |
| 22:6n3 Feed | m2 | 0.991 | 106 | Decis | 15.2 | 14.7 | 15.6 | 0 | 0 | 0 | -0.41 | -0.45 | -0.38 | 0.076 |
| 12:0 Mes | m1 | 0.978 | 63 | Strong | 0.28 | 0.24 | 0.32 | 0 | 0 | 0 | 0 | 0 | 0 | 0.893 |
| 14:0 Mes | m2 | 0.699 | 3 | Negl | 28.9 | 25.8 | 31.9 | 0 | 0 | 0 | -0.55 | -0.77 | -0.34 | 0.093 |
| 16:0 Mes | m1 | 0.659 | 4 | Subst | 76.5 | 70.5 | 82.4 | 0 | 0 | 0 | 0 | 0 | 0 | 0.106 |
| 18:0 Mes | m1 | 0.963 | 29 | Strong | 21.4 | 20.3 | 22.5 | 0 | 0 | 0 | 0 | 0 | 0 | 0.079 |
| 20:0 Mes | m1 | 0.964 | 33 | Strong | 2.6 | 2.43 | 2.77 | 0 | 0 | 0 | 0 | 0 | 0 | 0.561 |
| 16:1n7 Mes | m2 | 0.695 | 2 | Negl | 36.8 | 32.9 | 40.6 | 0 | 0 | 0 | -0.83 | -1.10 | -0.56 | 0.037 |
| 18:1n9c Mes | m4 | 0.807 | 5 | Subst | 220 | 187 | 250 | -28.3 | -54.4 | 2.2 | 5.83 | 3.83 | 7.81 | 0.997 |
| 20:1 Mes | m1 | 0.783 | 8 | Subst | 22.0 | 20.6 | 23.4 | 0 | 0 | 0 | 0 | 0 | 0 | 0.756 |
| 18:2n6c Mes | m4 | 0.517 | 1 | Negl | 79.5 | 68.0 | 90.8 | -10.6 | -21.4 | 0.6 | 1.97 | 1.27 | 2.67 | 0.851 |
| 18:3n3 Mes | m4 | 0.881 | 18 | Strong | 34.6 | 30.2 | 39.0 | -8.89 | -13.15 | -4.58 | 0.72 | 0.46 | 0.99 | 0.851 |

| 20:3n3 Mes | m4 | 0.722 | 3 | Negl | 3.1 | 2.77 | 3.41 | -0.843 | -1.157 | -0.53 | 0.07 | 0.05 | 0.09 | 0.998 |
| --- | --- | --- | --- | --- | --- | --- | --- | --- | --- | --- | --- | --- | --- | --- |
| 20:4n6 Mes | m2 | 0.582 | 2 | Negl | 4.3 | 3.84 | 4.80 | 0 | 0 | 0 | -0.11 | -0.15 | -0.08 | 0.849 |
| 20:5n3 Mes | m4 | 0.684 | 3 | Negl | 49.6 | 44.0 | 55.2 | 7.92 | 2.39 | 13.39 | -1.44 | -1.79 | -1.10 | 0.530 |
| 22:5n3 Mes | m2 | 0.820 | 5 | Subst | 22.4 | 20.1 | 24.8 | 0 | 0 | 0 | -0.55 | -0.71 | -0.38 | 0.275 |
| 22:6n3 Mes | m2 | 0.830 | 5 | Subst | 38.6 | 35.3 | 42.0 | 0 | 0 | 0 | -0.77 | -1.00 | -0.54 | 0.259 |
| 12:0 PI | m1 | 0.999 | 1357 | Decis | 0.04 | 0.03 | 0.04 | 0 | 0 | 0 | 0 | 0 | 0 | 0.140 |
| 14:0 PI | m1 | 0.546 | 2 | Negl | 3.64 | 3.07 | 4.22 | 0 | 0 | 0 | 0 | 0 | 0 | 0.420 |
| 16:0 PI | m1 | 0.699 | 2 | Negl | 12.3 | 11.2 | 13.4 | 0 | 0 | 0 | 0 | 0 | 0 | 0.315 |
| 18:0 PI | m1 | 0.846 | 5 | Subst | 4.12 | 3.87 | 4.37 | 0 | 0 | 0 | 0 | 0 | 0 | 0.284 |
| 20:0 PI | m1 | 0.979 | 61 | Strong | 0.48 | 0.40 | 0.56 | 0 | 0 | 0 | 0 | 0 | 0 | 0.016 |
| 16:1n7 PI | m2 | 0.652 | 3 | Negl | 5.80 | 5.02 | 6.58 | 0 | 0 | 0 | -0.16 | -0.21 | -0.10 | 0.951 |
| 18:1n9c PI | m4 | 0.733 | 5 | Subst | 5.05 | -0.55 | 10.69 | 8.92 | 3.39 | 14.39 | 1.78 | 1.44 | 2.13 | 0.849 |
| 20:1 PI | m2 | 0.469 | 2 | Negl | 0.91 | 0.38 | 1.43 | 0 | 0 | 0 | 0.09 | 0.06 | 0.13 | 0.788 |
| 18:2n6c PI | m4 | 0.627 | 2 | Negl | 0.38 | -1.49 | 2.25 | 2.88 | 1.04 | 4.71 | 0.62 | 0.51 | 0.74 | 0.910 |
| 18:3n3 PI | m2 | 0.687 | 2 | Negl | 0.91 | 0.22 | 1.60 | 0 | 0 | 0 | 0.19 | 0.14 | 0.24 | 0.808 |
| 20:3n3 PI | m2 | 0.932 | 23 | Strong | 0.13 | 0.06 | 0.20 | 0 | 0 | 0 | 0.02 | 0.02 | 0.03 | 0.880 |
| 20:4n6 PI | m2 | 0.976 | 59 | Strong | 1.4 | 1.31 | 1.47 | 0 | 0 | 0 | -0.03 | -0.03 | -0.02 | 0.793 |
| 20:5n3 PI | m2 | 0.840 | 8 | Subst | 9.2 | 8.05 | 10.38 | 0 | 0 | 0 | -0.24 | -0.33 | -0.16 | 0.916 |
| 22:5n3 PI | m2 | 0.924 | 16 | Strong | 2.4 | 2.21 | 2.65 | 0 | 0 | 0 | -0.05 | -0.07 | -0.04 | 0.218 |
| 22:6n3 PI | m2 | 0.790 | 5 | Subst | 7.1 | 6.54 | 7.62 | 0 | 0 | 0 | -0.12 | -0.16 | -0.08 | 0.107 |
| 12:0 LI | m1 | 1.000 | 18612 | Decis | 0.00 | 0.00 | 0.00 | 0 | 0 | 0 | 0 | 0 | 0 | 0.000 |
| 14:0 LI | m1 | 0.911 | 11 | Strong | 0.51 | 0.44 | 0.59 | 0 | 0 | 0 | 0 | 0 | 0 | 0.223 |
| 16:0 LI | m3 | 0.900 | 9 | Subst | 3.99 | 3.82 | 4.15 | 0.59 | 0.35 | 0.82 | 0 | 0 | 0 | 0.493 |
| 18:0 LI | m1 | 0.822 | 5 | Subst | 2.67 | 2.53 | 2.82 | 0 | 0 | 0 | 0 | 0 | 0 | 0.750 |
| 20:0 LI | m1 | 0.998 | 688 | Decis | 0.10 | 0.09 | 0.11 | 0 | 0 | 0 | 0 | 0 | 0 | 0.001 |
| 16:1n7 LI | m1 | 0.799 | 4 | Subst | 0.76 | 0.62 | 0.89 | 0 | 0 | 0 | 0 | 0 | 0 | 0.097 |
| 18:1n9c LI | m2 | 0.543 | 2 | Negl | 2.64 | -0.34 | 5.62 | 0 | 0 | 0 | 0.50 | 0.29 | 0.71 | 0.111 |
| 20:1 LI | m2 | 0.741 | 3 | Negl | 0.27 | -0.04 | 0.58 | 0 | 0 | 0 | 0.06 | 0.04 | 0.08 | 0.093 |
| 18:2n6c LI | m2 | 0.772 | 6 | Subst | 0.44 | -0.40 | 1.28 | 0 | 0 | 0 | 0.16 | 0.11 | 0.22 | 0.092 |
| 18:3n3 LI | m1 | 0.634 | 2 | Negl | 0.68 | 0.43 | 0.93 | 0 | 0 | 0 | 0 | 0 | 0 | 0.001 |

| 20:3n3 LI | m1 | 0.767 | 3 | negl | 0.21 | 0.13 | 0.28 | 0 | 0 | 0 | 0 | 0 | 0 | 0.025 |
| --- | --- | --- | --- | --- | --- | --- | --- | --- | --- | --- | --- | --- | --- | --- |
| 20:4n6 LI | m2 | 0.584 | 2 | Negl | 1.5 | 1.47 | 1.61 | 0 | 0 | 0 | -0.02 | -0.02 | -0.01 | 0.988 |
| 20:5n3 LI | m4 | 0.383 | 1 | Negl | 4.5 | 4.20 | 4.82 | 0.58 | 0.27 | 0.89 | -0.07 | -0.09 | -0.05 | 0.716 |
| 22:5n3 LI | m2 | 0.955 | 26 | Strong | 2.1 | 1.94 | 2.23 | 0 | 0 | 0 | -0.05 | -0.06 | -0.03 | 0.222 |
| 22:6n3 LI | m3 | 0.875 | 13 | Strong | 5.5 | 5.29 | 5.72 | 0.77 | 0.47 | 1.08 | 0 | 0 | 0 | 0.990 |
| SUM Feed | m2 | 0.919 | 12 | Strong | 181.3 | 176 | 187 | 0 | 0 | 0 | 2.90 | 2.52 | 3.27 | 0.795 |
| SUM Mes | m4 | 0.459 | 2 | Negl | 663.2 | 592 | 731 | -15.3 | -60.4 | 33.1 | 3.63 | -0.98 | 8.23 | 0.716 |
| SUM PI | m4 | 0.770 | 7 | Subst | 55.0 | 42.1 | 68.3 | 20.4 | 7.4 | 32.7 | 1.94 | 1.13 | 2.75 | 0.702 |
| SUM LI | m4 | 0.436 | 2 | Negl | 23.8 | 18.7 | 28.9 | 8.16 | 3.16 | 13.10 | 0.62 | 0.31 | 0.93 | 0.890 |

- *Explanations to the abbreviations are given in Table S2.*

***Table S8.*** *Sea lice counting express as sea lice/^cm2^, welfare indicators (WI) and relative scoring based on Kolarevic and Hoyle scoring systems.*

| **Fish size** | **Sea lice number/cm^2^ of fish area** | | | |  | **WI scored by Kolarevic et al.** | |  | **WI scored by Hoyle et al.** | |
| --- | --- | --- | --- | --- | --- | --- | --- | --- | --- | --- |
|  | Non-  motile | Chalimus | Motile | Adult female | Scale loss | Skin lesions | Eye damage | Cataract | Dorsal fin damage | Caudal fin damage |
| Small fish | 0 | 0 | 0,006104 | 0,007522 | 1.16 | 0.37 | 0.083 | 0.25 | 1.25 | 1.4 |
| Large fish | 0 | 0 | 0,004115 | 0,005844 | 0.95 | 0.55 | 0.208 | 0.25 | 1.25 | 1.28 |

***Figure S1a.*** *Photo taken during the pit tagging process of the experimental animals.*


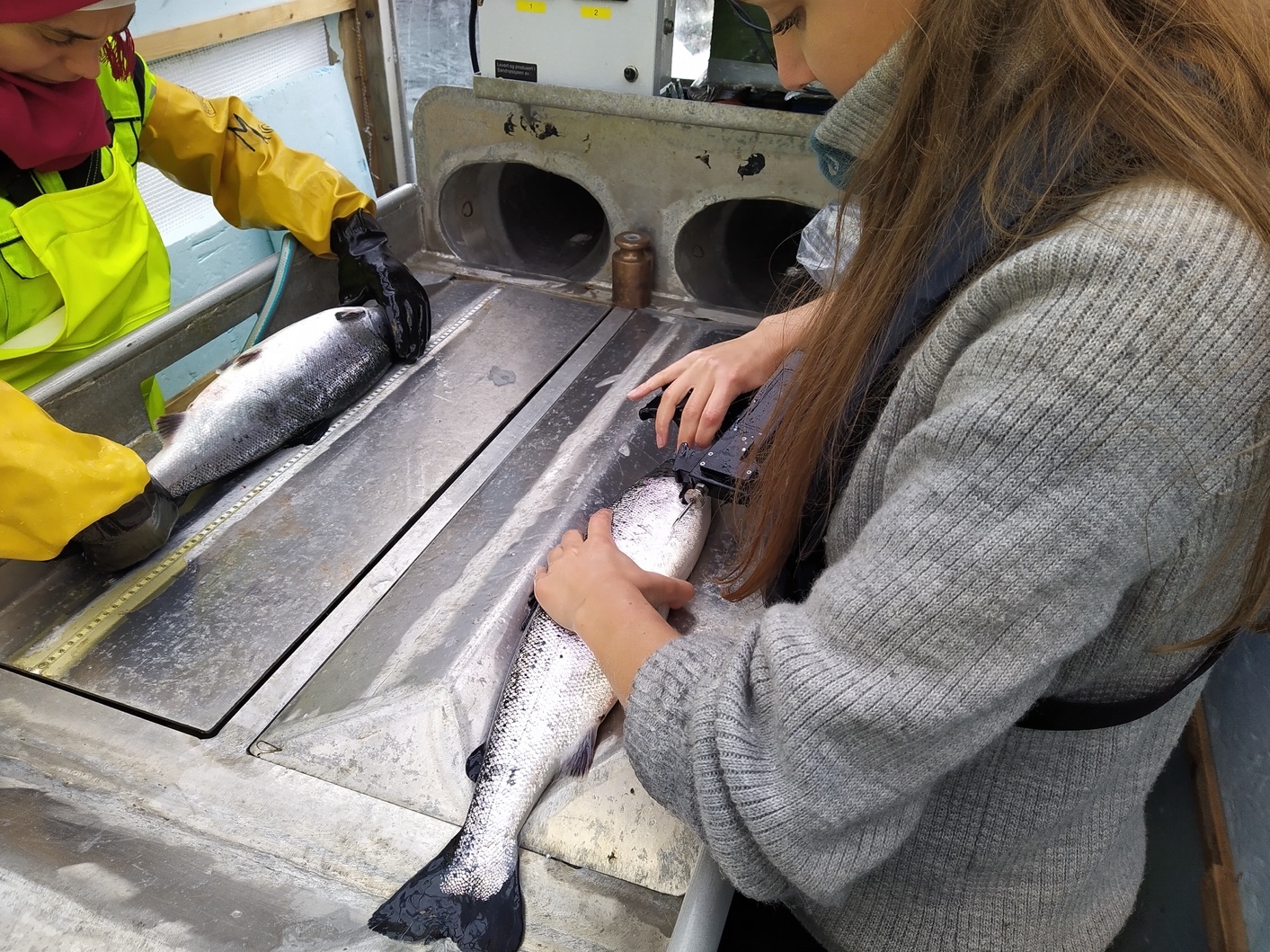


***Figure S1b.*** *Picture showing the experimental tanks at the LetSea facility.*


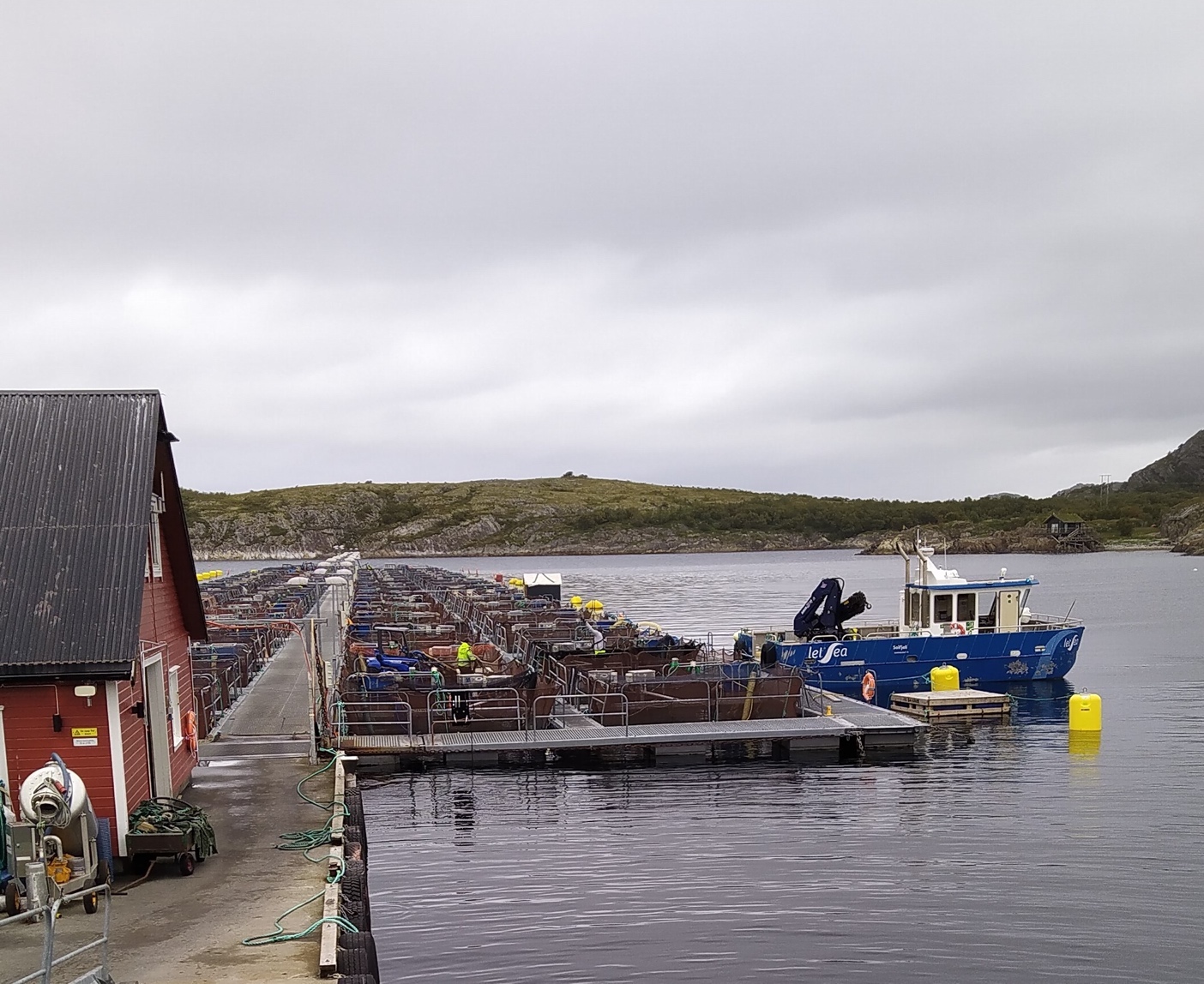


***Figure S1c.*** *Picture showing the experimental tanks at the LetSea facility.*


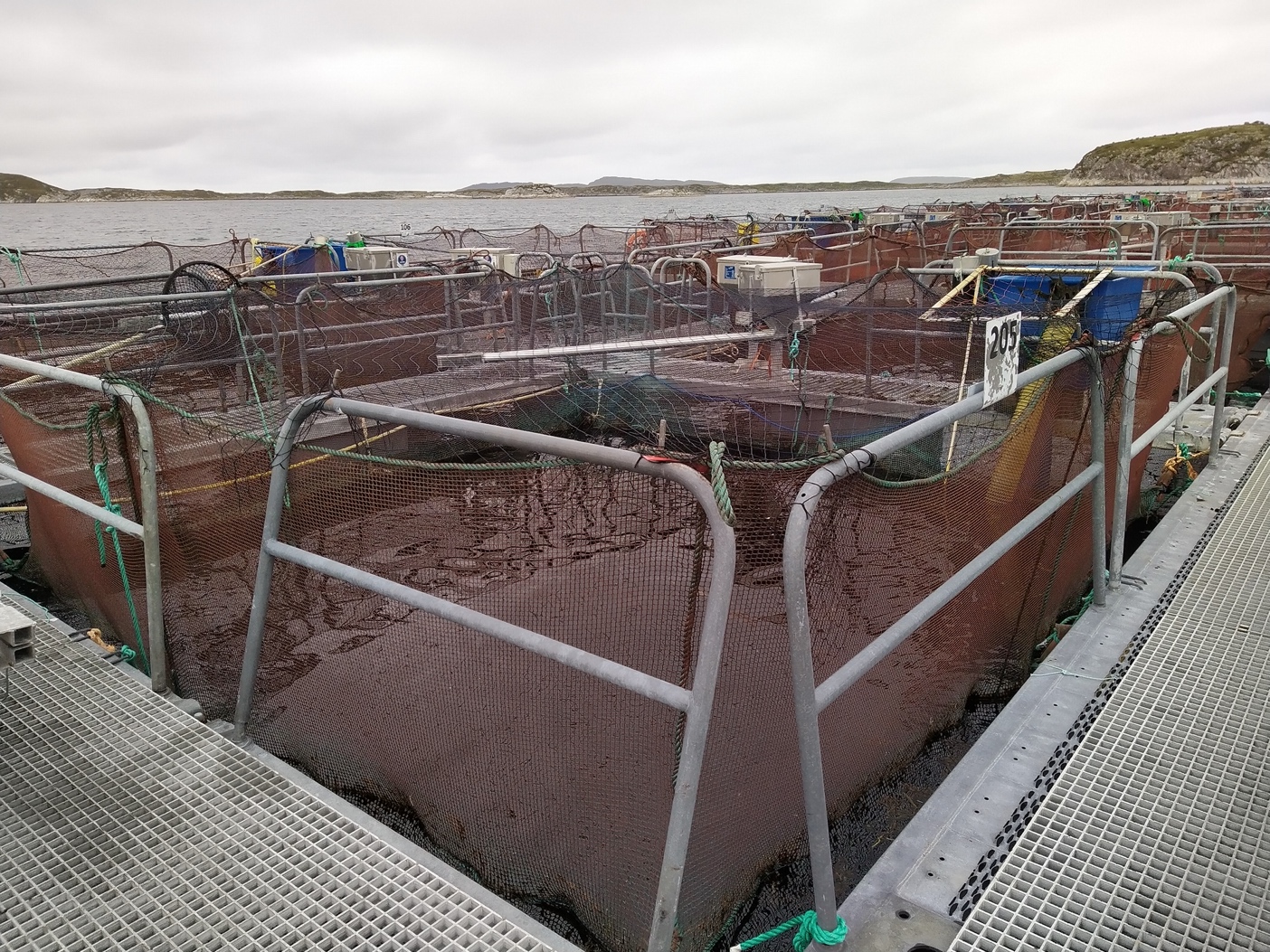


***Figure S2a.*** *Effects of the increasing dietary rapeseed oil level on content of saturated and mono-unsaturated fatty acids indicated on the left above the graphs, in absorbed fat, pyloric caeca (PI), mesenteric fatty tissue (Mes), and liver (LI) (Unit: g/kg feed or tissue), respectively for small (0) and large fish (1). The legend in the figure indicates whether fish size clearly affected the results and the best model selected for the data. For fatty acids not clearly affected by fish size, average cures are presented. For fatty acids showing significant effects of fish size, separate curves are shown. The curves show estimated regression on diet rapeseed oil level with indication of 95% credible intervals for the posterior means.*

, %

level

oil

Rapeseed


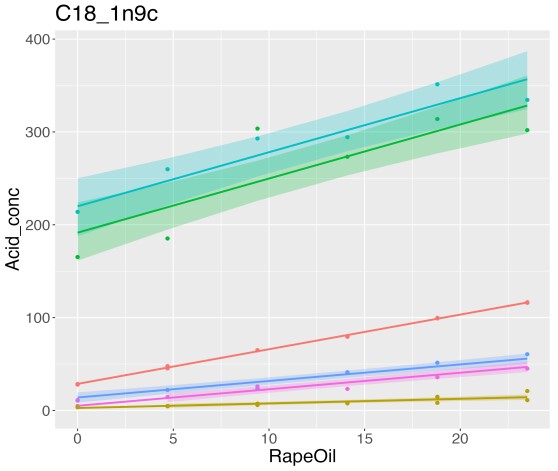

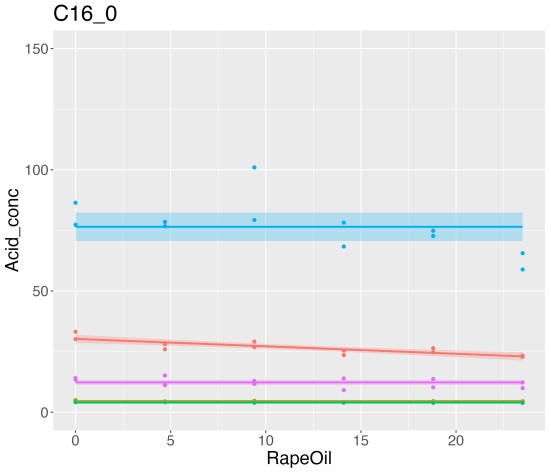

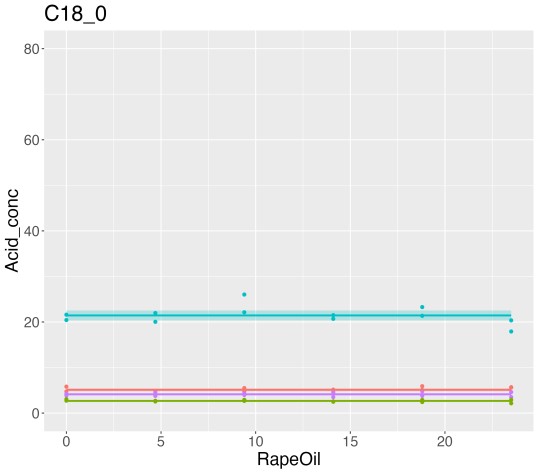

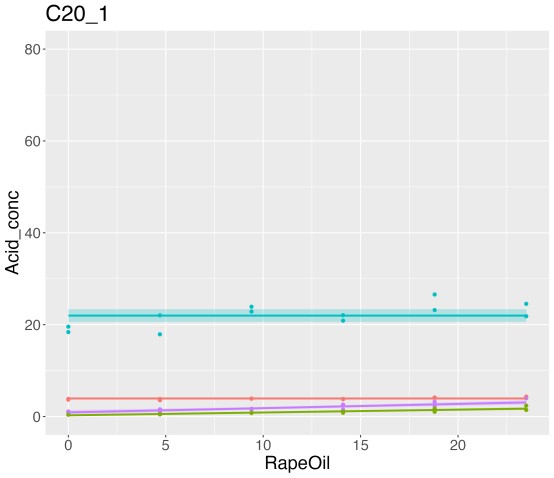

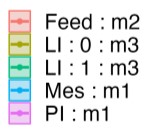

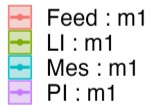

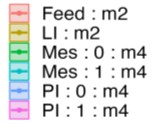

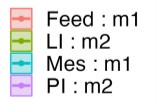

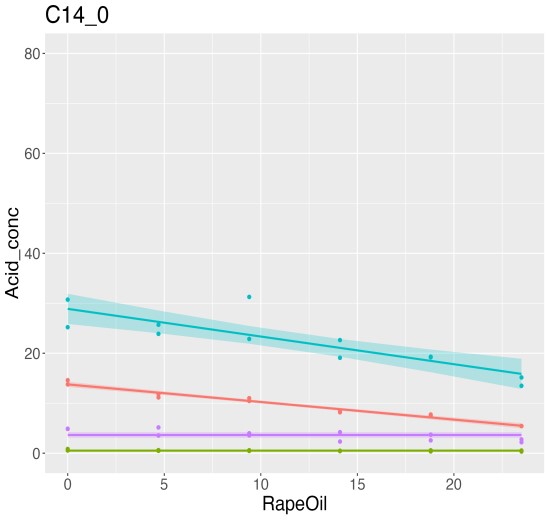

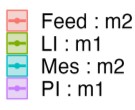

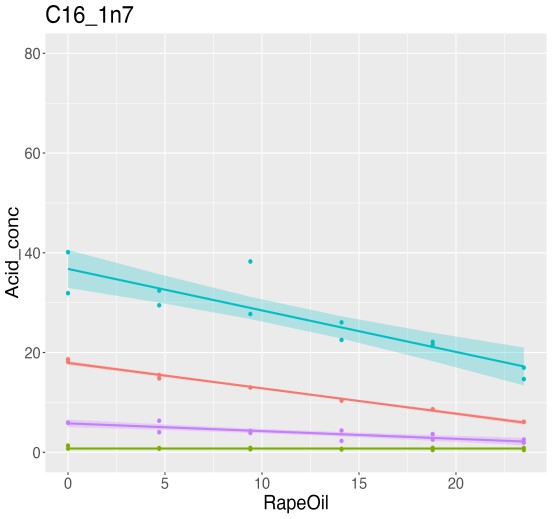

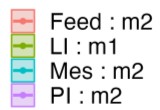


g/kg

g/kg

g/kg

g/kg

g/kg

Rapeseed

oil

level

, %

Rapeseed

oil

level

, %

Rapeseed

oil

level

, %

Rapeseed

oil

level

, %

Rapeseed

oil

level

, %

g/kg


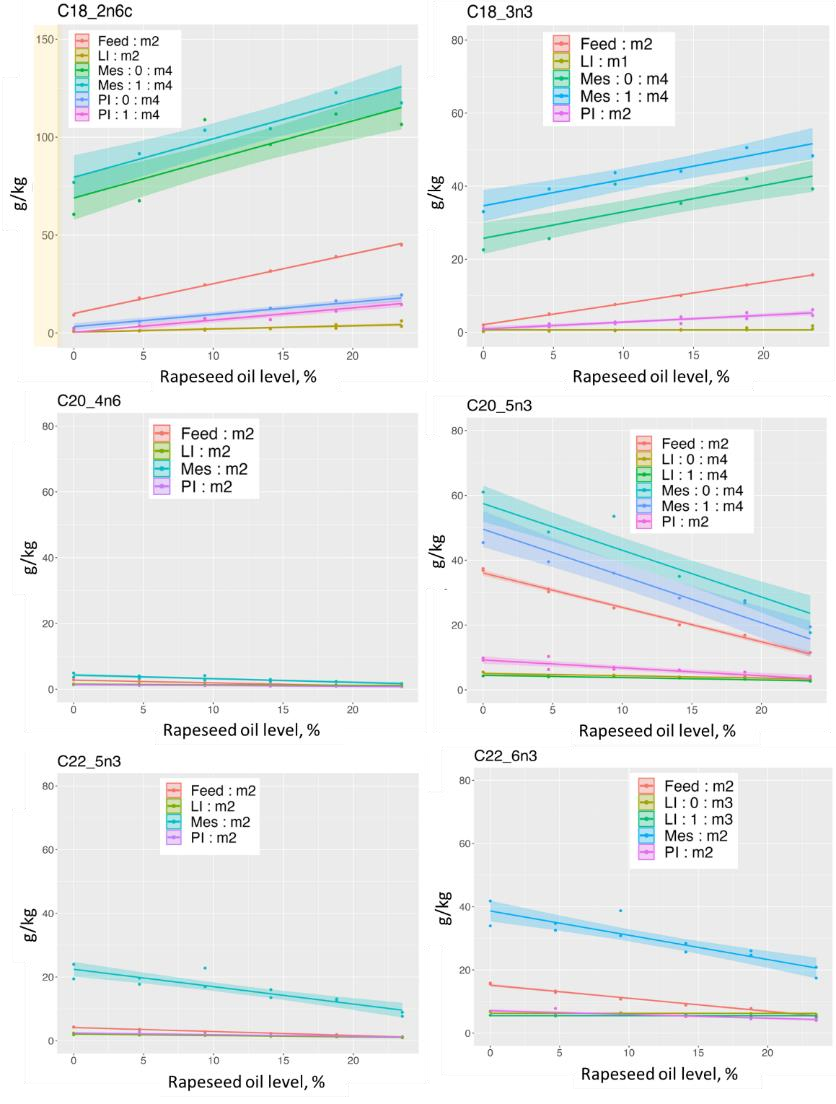


***Figure S2b.*** *Effects of the increasing dietary rapeseed oi level on content of n3 and n6 fatty acids indicated on the left above the graphs, in absorbed fat, pyloric caeca (PI), mesenteric fatty tissue (Mes), and liver (LI) (Unit: g/kg feed or tissue), respectively for small (0) and large fish (1). The legend in the figure indicates whether fish size clearly affected the results the best model selected for the data. For fatty acids not clearly affected by fish size, average cures are presented. For fatty acids showing significant effects of fish size, separate curves are shown. The curves show estimated regression on diet rapeseed oil level with indication of 95% credible* intervals for the posterior means.
